# A new leafminer on grapevine and *Rhoicissus* (Vitaceae) in South Africa within an expanded generic concept of *Holocacista* (Insecta, Lepidoptera, Heliozelidae)

**DOI:** 10.3897/zookeys.507.9536

**Published:** 2015-06-08

**Authors:** Erik J. van Nieukerken, Henk Geertsema

**Affiliations:** 1 Naturalis Biodiversity Center, PO Box 9557, NL-2300 RA Leiden, The Netherlands; 2 Research Associate, Department of Botany and Zoology, Stellenbosch University, Private Bag X1, Matieland, 7602 South Africa

**Keywords:** Host shift, leafminers, Vitaceae, viticulture, table grapes, *Rhoicissus*, *Cissus*, DNA barcode, genitalia, *Holocacista*, *Antispilina*, *Antispila*, Afrotropics, South Africa, Zimbabwe, India

## Abstract

A grapevine leafminer found recently in table grape orchards and vineyards in the Paarl region (Western Cape, South Africa) is described as *Holocacista capensis* **sp. n.** It has also been found on native *Rhoicissus digitata* and bred on that species in the laboratory. It is closely related to *Holocacista salutans* (Meyrick, 1921), **comb. n.** (from *Antispila*), described from Durban in KwaZulu-Natal, but widespread in southern Africa and a native leafminer of various Vitaceae: *Rhoicissus tomentosa*, *Rhoicissus digitata*, *Rhoicissus tridentata* and *Cissus cornifolia*. *Holocacista capensis* has been found on *Vitis vinifera* both in Gauteng and Western Cape, the earliest record being from 1950 in Pretoria. The initial host shift from native Vitaceae to *Vitis* must have occurred much earlier. The species is sometimes present in high densities, but hitherto no sizeable damage to the crops has been noted. The genus *Holocacista* Walsingham & Durrant, 1909, previously known from the single European grapevine leafminer *Holocacista rivillei* (Stainton, 1855), is expanded and redescribed and for the first time reported from Africa, East and South-East Asia and Australia. It comprises seven named species and at least 15 unnamed species. The following species are also recombined with *Holocacista*: transferred from *Antispilina*: South-African *Holocacista varii* (Mey, 2011), **comb. n.**, feeding on *Pelargonium*, transferred from *Antispila*: the Indian species *Holocacista micrarcha* (Meyrick, 1926), **comb. n.** and *Holocacista pariodelta* (Meyrick, 1929), **comb. n.**, both feeding on *Lannea coromandelica*, and *Holocacista selastis* (Meyrick, 1926), **comb. n.** on *Psychotria dalzelii*. We also remove the following from *Antispila*: *Heliozela anna* (Fletcher, 1920), **comb. n.** and *Heliozela argyrozona* (Meyrick, 1918), **comb. n.**, whereas the following Indian Vitaceae feeding species are confirmed to belong in *Antispila* s. str.: *Antispila argostoma* Meyrick, 1916 and *Antispila aristarcha* Meyrick, 1916. *Holocacista salutans* and *Holocacista varii* are redescribed and diagnosed against *Holocacista capensis* and other South African Heliozelidae. DNA barcodes are provided for 13 species of *Holocacista*.

## Introduction

The occurrence of leafmining Lepidoptera on cultivated grapevine has until recently been a minor economic problem, with just some damage in European viticulture by the native *Holocacista rivillei* (Stainton, 1855) (Camporese and Marchesini 1991; Alma 1995). In the last decades two cases have been reported of damage by grapevine leafminers introduced from North America into Europe: *Phyllocnistis vitegenella* Clemens, 1859 (family Gracillariidae) and *Antispila oinophylla* Van Nieukerken & Wagner, 2012 (family Heliozelidae) (Marchesini et al. 2000; van Nieukerken et al. 2012b). In Japan a native *Vitis*-feeding heliozelid infested cultivated grapevine: *Antispila uenoi* Kuroko, 1987 (Kuroko 1987; Ueno et al. 1987). In North America there are few reports of damage to cultivated grapes (e.g., Mcgiffen and Neunzig 1985), despite the rich Vitaceae-feeding fauna of heliozelids (van Nieukerken et al. 2012b). The recent infestations on cultivated grapevine with heliozelids were unexpected and in both situations required a new species to be described, despite the fact that Vitaceae as a hostplant for this family was a long-known fact, even recorded in the 18^th^ century (Godeheu De Riville 1750; van Nieukerken et al. 2012b).

In 1990 some cocoons of an unknown leafminer infesting vines grown on the experimental farm in Roodeplaat, Pretoria, were submitted by Miss S. Marais to HG. Together with moths reared in March 1998 from urban vines in Oudtshoorn (Western Cape), these were examined during a visit in March 1999 by Dr. Vári in Pretoria, who identified them tentatively as a possible species of *Antispila* Hübner, 1825, on the basis of the type of mine and external features. After Dr J. De Waal (Dow Chemicals) brought some infested vine leaves from a commercial table grape farm near Paarl, a visit by HG showed considerable infestations in local table grape orchards, ranging from Somerset-West to Paarl in the Western Cape. As the presence of the moth, especially on grapes destined for export, could pose serious economic problems, even though the infestation did not yet result in real damage to the crop, its potential future risks required further study of the identity of the leafminer and of its life history and infestation ecology.

The Heliozelidae are a small family of primitive Monotrysian moths, of which most species make leafmines as larvae, with 124 named species globally (van Nieukerken et al. 2012b). Taxonomically the family is poorly studied, with only the Japanese fauna relatively well known (Lee and Hirowatari 2013), and a recent description of the primitive South American genus *Plesiozela* Karsholt & Kristensen, 2003, based on the unpublished generic revision by Nielsen (1980) (Karsholt and Kristensen 2003). The discovery of two North American species that invaded Europe and attacked commercially grown crops led to a revival of taxonomic and phylogenetic studies, with extensive DNA barcoding (van Nieukerken et al. 2012b; van Nieukerken et al. 2013; Bernardo et al. 2015). One of the surprising results from these studies is the possibility that Vitaceae may form the ancestral hostplants of at least a large part of the family.

The African fauna of Heliozelidae is virtually unknown, only four species have been named to date, three of which are from South Africa. The fourth, *Antispila merinaella* Paulian & Viette, 1955, described from Madagascar (Paulian and Viette 1955), is misplaced and in fact belongs to Elachistidae (J. Minet personal communication), which also better fits the hostplant family Commelinaceae, on which several species of the genus *Elachista* Treitschke, 1833 occur (Kaila 2011). The South African species are *Antispila argyrozona* Meyrick, 1918, *Antispila salutans* Meyrick, 1921, both with unknown host (Kroon 1999; Vári et al. 2002) and the recently described *Antispilina varii* Mey, 2011, reared from leafmines on *Pelargonium* L.’Hérit. in the Western Cape (Mey 2011).

The late Lajos Vári (Pretoria) devoted a large part of his life to the study of leafmining Lepidoptera of southern Africa, which he extensively collected and reared between 1950 and 2007 (Kroon 2011). He only published on a limited part of this fauna, mainly Nepticulidae and Gracillariidae (Vári 1955; 1961; 1963), but did not describe the Heliozelidae that he collected. In fact, he had already discovered a *Vitis*-feeding heliozelid in 1950 in Pretoria as appears both from his notebooks and collection in Pretoria, but another record on *Vitis* L. has also been published somewhat hidden in a list as *Antispila* sp. (Kroon 1999). By studying Vári’s collection, it soon appeared that a small but diverse fauna of heliozelids is present in southern Africa, with the majority feeding on native Vitaceae. As observed before (van Nieukerken et al. 2012b), many species placed in *Antispila* probably do not belong to that genus, which can be regarded as a dumping ground for heliozelids with bright spots and fascia (van Nieukerken et al. 2012b). This is also true for the Southern African species, the majority of which belong to the previously monotypic genus *Holocacista* Walsingham & Durrant, 1909, and only one of the unnamed species belongs in *Antispila* s. str. Although a revision of the South African fauna would be desirable, we here limit ourselves to the identity and taxonomy of the grapevine miner, which will be described as a new species, and compared with *Holocacista salutans* (Meyrick, 1921), comb. n. and *Holocacista varii* (Mey, 2011), comb. n., both now re-described. We also describe its biology, provide DNA barcodes, and discuss a potential hostplant shift. Because the genus *Holocacista* previously only comprised the type species *Holocacista rivillei*, the genus is diagnosed and re-described here as well. In Appendix A we provide brief notes on other South African Heliozelidae.

## Material and methods

### Material

Larvae, cocoons and adults of *Holocacista capensis* were collected in various table grape orchards, vineyards and weedy growth of grapevine in the Paarl region, several other localities in the Western Cape and at Roodeplaat, north of Pretoria. We selected the Holotype from our recent reared material, so that the name unequivocally refers to the grapevine leafminer, and to couple its morphology with DNA data. Although the description is based on multiple specimens, we refrain from selecting paratypes as they have no name-bearing function (International Commission on Zoological Nomenclature 1999). The many specimens reared by us will be divided among the collections in Leiden (Naturalis), Stellenbosch (USEC), Pretoria (ARC-PPRI and TMSA), and Cape Town (Iziko).

The extensive collections in the Ditsong Museum of Natural History (formerly Transvaal Museum, Pretoria) formed the basis for most of the present taxonomic studies, together with our newly collected material. The majority of the Pretoria material was collected and reared by Lajos Vári. The material comprises three important sources:

1. *Dry pinned collection of adults.* These are usually rather poorly labelled, with only a locality name (often in capitals), sometimes followed by a second indication of the locality, the handwritten date of emergence of the adult, sometimes the name of the collector followed by an Ac. no. [Accession number] followed by a handwritten number. There are no further details, no hostplant names nor original collection dates (unless it has been collected as an adult). In the collection, which is organised in unit trays, often a single example of a leaf with mines is pinned next to the reared moths, usually with a handwritten number on the leaf.
2. *Herbarium of leafmines*. The original collection was stored in simple open envelopes with a number, sometimes with a small label inside, but often not. The majority of this collection has later been mounted by collection staff onto herbarium sheets, partly glued to the sheet and mounted with white strips. The Ac. numbers are also written on these sheets, sometimes with additional information.
3. *Notebooks of Lajos Vári.* This is an essential source to reconstruct the hostplant data, detailed localities and collection data. They also give insight in Vári’s concepts of the species he collected; he often gave manuscript names, sometimes changed subsequently and referring to earlier numbers when he considered hostplants or moth species to be identical. The Ac. numbers from the labels provide access to the notes; these numbers only refer to Vári’s material and are not a general numbering for the museum.

The data we present under material and in Suppl. material 1 are the label data, supplemented with details from these notebooks.

For understanding the generic composition of the genus *Holocacista*, we studied several Heliozelidae available to us, and examined a number of Indian species described by Meyrick, that were potential candidates to belong to this genus.

Details on all studied specimens, including those sequenced, are given as an Excel sheet in Suppl. material 1.

### Abbreviations for depositories etc.

ARC-PPRI Agricultural Research Council – Plant Protection Research Institute, Pretoria, South Africa

BMNH Natural History Museum, London, UK [for slides only the abbreviation BM is used]

EvN E.J. van Nieukerken [for slide and rearing numbers]

HG Personal collection of H. Geertsema, Stellenbosch, South Africa

ISAM Iziko South African Museum, Cape Town, South Africa

JCK J.C. Koster [for slide numbers]

MHUB Museum für Naturkunde, Berlin, Germany

RMNH Naturalis Biodiversity Center, Leiden, The Netherlands

TMSA Ditsong National Museum of Natural History (formerly Transvaal Museum), Pretoria, South Africa [for slides only the abbreviation TM is used]

USEC University Stellenbosch Entomology Collection, Stellenbosch, South Africa.

### Rearing

Leaves with active mines were cut to smaller pieces and placed in small plastic containers for rearing. After the shields had been exscinded and the larvae had left the mines, the leaves were removed to avoid moulding, and examples of leafmines were dried in a plant press. Often the larvae in their shields needed some ‘assistance’ with forceps in order to remove them from the leaf fragments, probably because the low turgor pressure in the leaf fragments made it difficult to exit in the natural fashion, where a higher turgor pressure facilitates the release of the shield. The larval shields and the resulting cocoons were kept with some paper tissue in closed containers, and a little moisture added from time to time until emergence of the moths. Emergence from cocoons kept in this way was abundant.

### Morphology

Methods for preparation of the genitalia follow van Nieukerken et al. (2012b). Male genitalia and wings were stained with phenosafranin and mounted in Euparal, often after studying material first in glycerine; for some specimens, genitalia were stored in glycerine in small vials; female genitalia were stained with Chlorazol black. Venation was studied in descaled wings that were stained with phenosafranin, after cleaning in ethanol 70% and embedded in Euparal. Whole body preparations of adults were prepared from specimens in ethanol, largely following Lee and Brown (2006) and also stained with phenosafranin.

Morphological terminology follows other recent treatments of Heliozelidae (Karsholt and Kristensen 2003; van Nieukerken et al. 2012b), the generic description follows the format set for *Plesiozela* (Karsholt and Kristensen 2003), and partly also follows Nielsen’s unpublished thesis (Nielsen 1980). We also include information extracted from Nielsen’s manuscript description of *Holocacista*, compared with our material and more species. Compared to the 2012 treatment (van Nieukerken et al. 2012b), the labelling of wing veins has slightly been changed; also since then we realised that antennal flagellomeres of Heliozelidae have two annuli of scales, and thus the number of antennal segments given then was incorrect and about twice the actual number (van Nieukerken et al. 2012b; Bernardo et al. 2015).

Photographs of mounted moths were prepared using an AxioCam digital camera attached to a motorized Zeiss SteREO Discovery V12, using the Module Extended Focus in the Carl Zeiss AxioVision software to prepare a picture in full focus from a Z-stack of about 10 to 25 individual photos. Leafmines and live adults were photographed with a similar camera on a manually operated Zeiss Stemi SV11 stereo-microscope, without extended focus, or with extended focus prepared from just a few exposures. Genitalia and wing slides were photographed with a similar camera on a manually operated Zeiss Axioskop H, usually with just a single exposure. Leafmines were examined and photographed with dark field illumination. Field photographs were taken with a Canon EOS camera.

Photographs were edited with Adobe Photoshop®, avoiding any change to the real object, but backgrounds were cleaned from excess debris; also some protruding scales were digitally removed from the denuded wings in Figs 29–36. Photos of wing venation were taken in sections, and combined with the photomerge tool in Photoshop. This was also done for some large leafmines. Drawings of genitalia were prepared using a drawing tube attached to the Zeiss Axioskop H. Some photos and drawings are given here as mirror image, in order to get all figures in a comparable orientation.

Host plant names of South African plants follow Germishuizen and Meyer (2003) and identifications of Vitaceae were checked and updated with Palgrave and Palgrave (2002). Other plant names were checked with The Plant List (2013).

### Molecular analysis

DNA was extracted non-destructively from larvae in ethanol 96% or adult abdomens (Knölke et al. 2005). Larvae were cut with a scalpel at three positions: one in the anterior region behind the head, one in the middle region of the body and one in the posterior region. One side of the body was cut to save the larval cuticle. After lysis, larval pelts were temporarily kept in ethanol 70% to allow future mounting on slide, abdomens and genitalia were dissected and mounted on slides or stored temporarily in glycerine. Extraction was carried out with the Qiagen DNEasey Blood and Tissue kit.

A 665 bp or a 658 bp fragment of the mitochondrial CO1 gene, the DNA barcode, was amplified. PCR conditions and primers are described in our earlier studies (van Nieukerken et al. 2012a; van Nieukerken et al. 2012b; Doorenweerd et al. 2015). Sequencing was outsourced to Macrogen Corporation, Amsterdam or BaseClear B.V., Leiden.

The sequence data generated and used in this study have been deposited in the public BOLD dataset “Holocacista leafminers [DS-HOLOCAC]” [http://dx.doi.org/10.5883/DS-HOLOCAC] and GenBank, they are listed with all details in the table with all studied specimens (Suppl. material 1). All specimens used for DNA barcodes and larvae stored as tissue samples in ethanol 96% and kept in a minus 80 freezer, received a RMNH.INS registry number, irrespective whether the original specimen belongs to the Naturalis collections or not. EvN 4-digit Genitalia slide numbers translate into a 5-digit RMNH.INS. number by adding 20,000; thus genitalia slide EvN4622 is associated with specimen and DNA extract RMNH.INS.24622.

Neighbor joining trees were prepared with the tools provided on the Boldsystems website (Ratnasingham and Hebert 2007), using “Pairwise Distance” as distance model. Further model based phylogenetic analyses were not carried out, since the CO1 gene does not have sufficient information for phylogeny. Analyses of several genes for the whole family are currently being studied and will be published elsewhere.

### Field observations

Monthly field visits to the infected vineyard at De Akker (Paarl South) were conducted between February 8 and May 11, 2012, and again from November 2012 to May 2013. From 2013 the vineyard at De Heuvel estate was also visited regularly. At monthly intervals, leaf samples (n = 100) were randomly collected from selected vineyards. Leaves were classified into three size groups, viz. large (older), medium and small (young), and numbers of mines (developing, containing shields or larvae, or holes) were noted to determine any preference for the major table grape cultivars and to determine variation in the population density throughout the grape season.

During 23–25 March 2012, a survey of vineyards between Worcester and Swellendam was carried out, as well as a search for signs of heliozelid leafmines on *Rhoicissus* Planch. species present on the periphery of indigenous temperate forest in the Swellendam district. Search for those mines was repeated in 2013 and 2014, but no live moths were found or reared.

## Results

### Identification

Moths reared from the grapevine leafmines were compared with other available adult Heliozelidae, and the similarity with European *Holocacista rivillei* became immediately apparent, particularly by the remarkable curved spine on the phallus, but also by venation and colour pattern. Comparison with the three named South African species also showed similarity to the species *Antispila salutans* Meyrick, 1921 and *Antispilina varii* Mey, 2011, and for some time we used the name “*Holocacista salutans*”. However, a detailed analysis of genitalia and comparison with material reared by Lajos Vári, made it clear that our grapevine feeder was neither of these species, but closely resembled a single population reared by Vári from leafmines on *Rhoicissus digitata* (L.f.) Gilg. & M.Brandt. On the basis of the morphological similarity with *Holocacista rivillei* (genitalia and wing venation) and an initial molecular phylogeny (data not presented here), it was clear that the new species belongs to the same clade as *Holocacista rivillei* and not to the clade that comprises either the type species of *Antispila* Hübner, [1825] (viz. *Antispila metallella* ([Denis & Schiffermüller], 1775) or the clade with the type species of *Antispilina* Hering, 1941 (*Antispilina ludwigi* Hering, 1941). Since this clade comprises several moths with similar morphology, we enlarge the genus *Holocacista* here and describe the grapevine leafminer as a new species. We therefore also redefine the genus *Holocacista* here and newly combine several species with it.

## Taxonomy

### Genus Holocacista

Taxon classificationAnimaliaLepidopteraHeliozelidae

Walsingham & Durrant

Holocacista Walsingham & Durrant, in Walsingham 1909: xxix. Type species (by original designation and monotypy): *Elachista rivillei* Stainton, 1855: 89.
Holocacista ; Nielsen (1980): 105 [re-description in unpublished thesis].

#### Differential diagnosis.

Very small moths, wingspan between 3 and 5 mm, usually with a pattern of metallic-silvery spots, but in some species not metallic, typically comprising a dorsal and costal spot at 1/4 sometimes united into a fascia and a postmedial fascia, which also may be broken into two spots. In some species part of this pattern is absent. Males never with androconial scales or hair-pencils. Separated from externally somewhat similar *Antispila* species by the reduced venation (Figs 29–34); in *Antispila* the discoidal cell is present and more veins are retained (Fig. 35); also most *Antispila* species are larger and have more antennal segments. Separated from most *Heliozela* species by more extensive colour pattern, the few *Heliozela* that do have more spots can be separated also by the venation with discoidal cell (Fig. 36), also *Heliozela* species have more antennal segments. Species of *Antispilina* and some in the “Antispila” ampelopsifoliella group have a very similar venation and are more difficult to separate; most *Holocacista* differ in the male genitalia by the usually long appendix on the phallus, moreover they have a small epiphysis, which is absent in the other genera with reduced venation.

#### Description.

**Adults.** Very small moths, forewing length ca. 1.5–2.5 mm (wingspan ca. 3–5 mm).

*Head* (Figs 21–24). Almost oval in outline. Eyes in latero-ventral position, ventral margin not reaching lower margin of head. Eye phragma narrow, weakly melanised. No sutures present. Anterior tentorial arms very slender, prominently curved laterally before converging towards frons. Vestiture comprising lamellar scales, firmly appressed on head, in dry specimens scales on vertex sometimes raised, probably an artefact as a result of drying. Mouthparts: labrum narrow, pilifers absent. Mandibles small, as long as broad, relatively well sclerotised (Fig. 23). Maxilla with galea well developed and longer than head; maxillary palp reduced to a single segment. Labial palp well developed, 3-segmented, drooping, slightly shorter than head capsule; distal segment from slightly longer to 1.5× second segment; depression for Organ von Rath not seen. Antenna (Fig. 24) ca. half length of forewing with 14–20 segments [best counted in denuded specimens on slides], no sexual dimorphism. Scape and pedicel of equal length, slightly shorter than flagellomeres. Flagellomeres cylindrical, longer than wide, each with two annuli of scales, often differently coloured, resulting in visible dark and pale rings from above. Pecten present, but not easily visible; Nielsen (1980) counted 4–6 hairs in *Holocacista rivillei*.

*Thorax*. Vestiture of appressed lamellar scales, usually concolorous with ground colour of forewings. Foreleg with small but distinct epiphysis of about 36–48 μm in *Holocacista rivillei* (Fig. 26) to 50–82 µm in *Holocacista capensis* (Fig. 25), distinctly smaller than in *Heliozela* (Fig. 27) (150–165 μm in two measured European specimens) and without the microtrichia along the inner side, which probably serve as antennal cleaning apparatus. In *Antispila*, *Antispilina* (Fig. 28) and *Coptodisca* epiphysis completely lacking.

*Wings*. Male retinaculum a series of 7–12 hook-shaped bristles, arising from a thickened serrate portion of Sc. Frenulum in male a strong curved bristle (e.g., Fig. 30), in female two bristles present (Figs 29, 34); no pseudofrenular bristles in male. Humeral field with scattered microtrichia, otherwise restricted on wing membrane to area just posterior of retinaculum, arranged in longitudinal rows. Scale sockets regularly spaced, not in distinct rows.

*Venation* in forewing (Figs 29–34) with Sc to middle of costa. R unbranched, a separate vein, to costa. Rs with M and CuA, ending in 3–4 branches, interpreted as Rs1+2 to costa, Rs3+4 to termen, M and CuA to dorsum; in one species, *Holocacista* sp. *_Terminalia_SA*, tentatively placed here, Rs+M+Cu even more reduced, with only 2 branches. Hindwing with Sc+R to costa, Rs+ M with 2–3 branches, Rs to costa, 1 or 2 branches of M to termen and dorsum; CuA a separate vein to dorsum.

**Figures 1–4.**
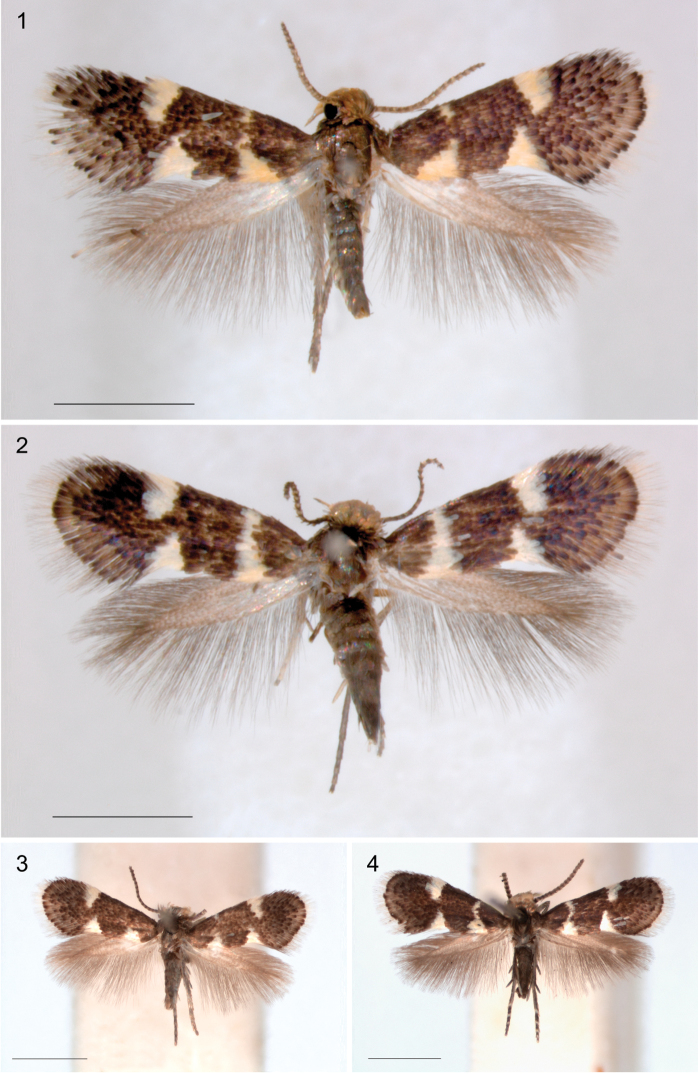
*Holocacista* species, adult habitus. **1–3** *Holocacista capensis*: **1** Male holotype, RMNH.INS.24622 **2** Female, Western Cape, Paarl, RMNH.INS.24624 **3** Male, Western Cape, Wilderness, reared from *Rhoicissus digitata*, Genitalia slide EvN4381 **4** *Holocacista salutans*, male, Kwazulu-Natal, Umhlanga Rocks, reared from *Rhoicissus revoilii*, Genitalia slide EvN 4383. Scales 1 mm.

**Figures 5–12.**
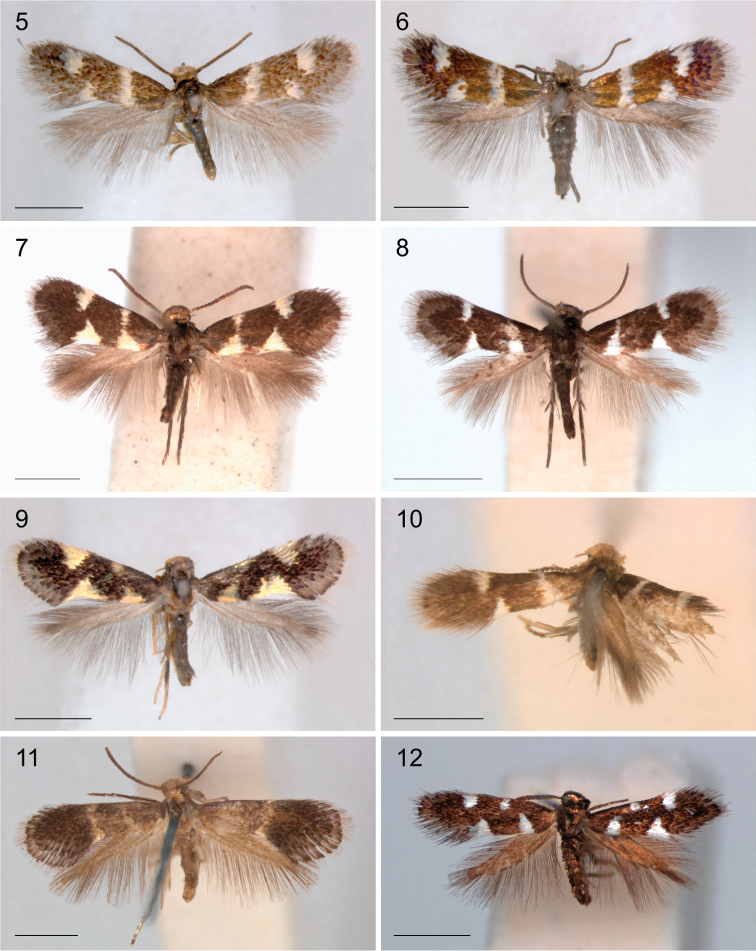
*Holocacista* species, adult habitus. **5, 6** *Holocacista varii*, Western Cape, Table mountain NP **5** Male, RMNH.INS.24623 **6** Female RMNH.INS.24625 **7** *Holocacista* sp. *Rhoicissus_tridentata*, male, Zimbabwe, Mt. Selinda, Genitalia slide EvN4385 **8** *Holocacista* sp. *Cissus_integrifolia*, male, Zimbabwe, Lundi, Genitalia slide EvN4387 **9** *Holocacista rivillei*, male, Italy, Borghetto, RMNH.INS.24626 **10** *Holocacista micrarcha*, male **11** *Holocacista selastis*, male **12** *H. Leea_Borneo*, male, Indonesia, Kalimantan Timur, Gunung Lumut, RMNH.INS.24158. Scales 1 mm.

**Figures 13–20.**
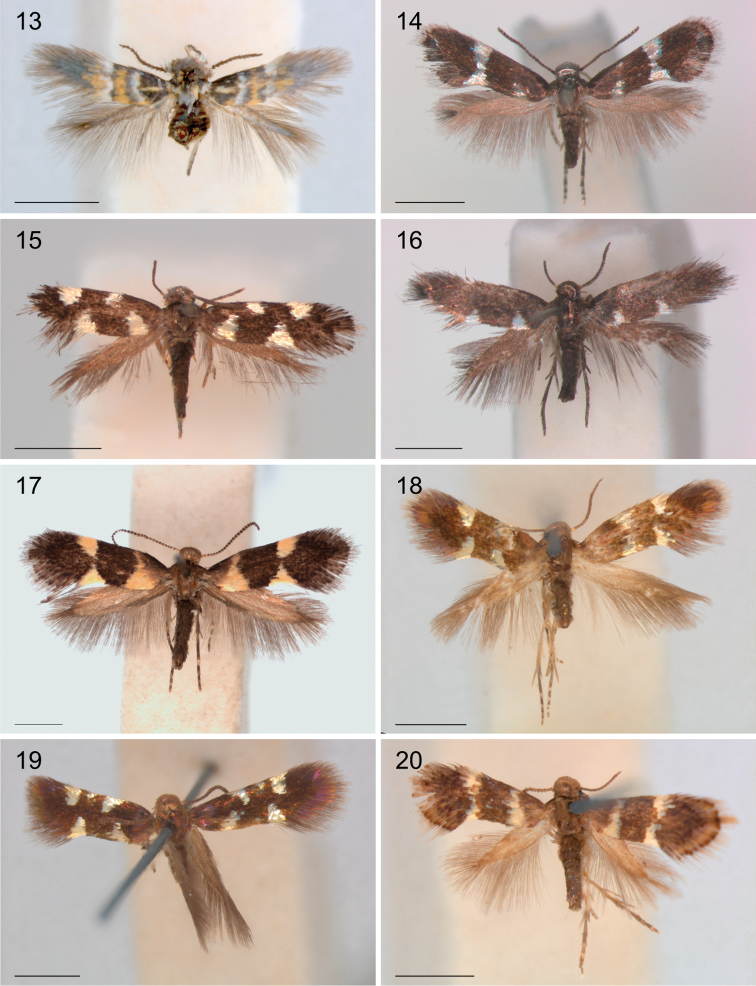
Heliozelidae species, adult habitus. **13** *Holocacista* sp. *Dyerophytum_*UAE, Male, UAE, Fujairah, RMNH.INS.24628 **14** *Holocacista* sp. *Psychotria_*Australia, male, Australia, Queensland, RMNH.INS.24367 **15** *Holocacista* sp. *Lasianthus_*Borneo, female, Indonesia, Kalimantan Timur, Gunung Lumut, RMNH.INS.24159 **16** *Holocacista* sp. *Impatiens_*Vietnam, male, Vietnam, Cuc Phuong NP, RMNH.INS.24361 **17** *Antispila* sp. *Rhoicissus_*SA, male, South Africa, Limpopo, Louis Trichard, Genitalia slide EvN4379 **18** *Antispila argostoma*, male, India **19** *Antispila aristarcha*, female, India **20** *Heliozela anna*, female, India. Scales 1 mm.

*Wing pattern* (Figs 1–16). On forewing typically comprising a pair of opposite pale, often metallic spots at 1/3 and a similarly coloured fascia or opposite spots at 2/3 on a dark background, brown to black, or brassy and shining. Variation exists in relative size, whether spots are joined to a fascia, or fascia is broken, or spots may be absent (e.g., Figs 11, 14, 16). Many species show sexual dimorphy in pattern, with females having more or larger pale elements than male. Only a single species from Arabian Peninsula has a different pattern with much yellow, probably as an adaptation to the desert habitat (Fig. 13). A fringe line often available, with fringe scales pale. Hindwings uniform grey. Androconial scales absent in all species examined.

*Pregenital abdomen*. Abdominal sclerites weakly sclerotised. Anterior sternum II subtriangular, free.

*Male genitalia*. Vinculum (S IX) very long, anteriorly often reaching beyond anterior margin of segment VI, almost cylindrical; tegumen (TIX) narrow, usually with a medial posterior process, probably a composite structure with uncus. Gnathos absent. Valva rather narrow, with stalked pectinifer halfway to inner margin, pecten comprising 6–12 blunt sensilla; transtilla typically with medial anterior projection, sublateral processes long. Phallocrypt (manica) with some to many strongly-sclerotised conical spines, often arranged in an asymmetric fashion, or with many smaller spines. Phallus outer tube often with remarkable ventrally-curved appendix on phallus, or appendices of different sizes and shapes. Juxta present and often bilobed or reduced to narrow ventral process.

*Female genitalia*. SVIII pointed, T VIII deeply indented. Oviscapt with few lateral cusps. Anterior and posterior apophyses subequal in length. Spermathecal papilla usually with circular sclerotisation. Ductus spermathecae with many coils.

*Larva.* Larvae yellow or whitish, usually with darker head capsule. Larva of *Holocacista rivillei* described in detail by Grandi (1931) and Marchi (1956). Head prognathous, legs and prolegs absent, but paired ambulatory calli on T2 and 3 (ventral and dorsal) and fused ventro-medial – calli on A3–6. Larvae with four feeding instars and a fifth non-feeding instar that constructs the case in which it pupates.

#### Biology.

**Hostplants.** Several species feed on Vitaceae and Rubiaceae, a few species on Anacardiaceae, and single species each on Balsaminaceae, Dilleniaceae, Geraniaceae and Plumbaginaceae. A species feeding on Combretaceae is tentatively added, but this requires confirmation.

**Life history.** Eggs are inserted in leaf tissue, often near a vein or leaf margin. All species construct leafmines (Figs 70–75, 79, 83–93), usually starting as a narrow linear mine, later usually widening into a blotch, or sometimes remaining an irregularly wide gallery, and cut out an oval shield, comprising the epidermal layers, during the penultimate instar. Frass is deposited in a central line in the mine or filling the mine, later often scattered in the blotch or pushed by the larva to one side. The shields (Figs 76–78), later forming the cocoons, are more or less flat, without the raised ridge that is characteristic for *Antispila*. They attach this cocoon to any surface (trunks, leaves, leaf litter, etc.) where the non-feeding final instar larva pupates. Adults are usually day flying (Figs 80–82), and rarely come to light.

#### Distribution.

Mainly Old World tropics and subtropics: Afrotropical, Oriental and Australian regions, north to Taiwan and southern Europe (type species). Some DNA barcodes suggest that the genus also occurs in South and Central America, but no adults have yet been studied from this area.

#### Composition.

The species listed in the checklist below, both named ones and unnamed ones, share the external and venation characters described above, and those dissected also the male genitalia characters. Those species that we have been able to sequence form a well-supported clade in a phylogenetic analysis (both Bayesian and Maximum Likelihood) of the Heliozelidae based on four genes (unpublished study in progress), as part of a larger clade of genera with reduced venation (including also *Antispilina* Hering, 1941, *Coptodisca* Walsingham, 1895 and the “Antispila” ampelopsifoliella group). While checking several Indian species described by Meyrick, we could also change the following generic assignment: *Heliozela anna* (Fletcher, 1920), comb. n. (from *Antispila*, feeding on Myrtaceae) (Figs 20, 36), whereas the following remain in their original genus: *Antispila argostoma* Meyrick, 1916 (Figs 18, 35) and *Antispila aristarcha* Meyrick, 1916 (Fig. 19), both feeding on Vitaceae. While we assign an unnamed species feeding on *Impatiens* from Vietnam to *Holocacista* here (Figs 16, 34), the Indonesian *Microplitica metadesmia* (Meyrick, 1934) that likewise feeds on *Impatiens*, has a completely different venation, more similar to *Heliozela* Herrich-Schäffer, 1853. Pending further study of this species and the type species of *Microplitica* Meyrick, 1935 (*Microplitis desmophanes* Meyrick, 1922), we leave it in *Microplitica* for now.

In the checklist below we provide the original genus in brackets, type locality, and the hostplant of the types. The species are listed geographically, first the named ones, then the unnamed ones.

#### Checklist

Palearctic species

*Holocacista rivillei* (Stainton, 1855): p. 89 (*Elachista*)

Malta, *Vitis vinifera* L. [type species]

African species

*Holocacista capensis* van Nieukerken & Geertsema, sp. n.

South Africa, Western Cape, Paarl, *Vitis vinifera* L.

*Holocacista salutans* (Meyrick, 1921): p. 108, comb. n. (*Antispila*)

South Africa, [Kwazulu Natal], Durban, [*Rhoicissus* sp.]

*Holocacista varii* (Mey, 2011): p. 156, comb. n. (*Antispilina*)

South Africa, Western Cape, Cape Town, *Pelargonium cucullatum* (L.) L’Hérit.

Asian species

*Holocacista micrarcha* (Meyrick, 1926): p. 261, comb. n. (*Antispila*)

India, [Karnataka], Karwar, *Lannea coromandelica* (Houtt.) Merr. (=*Odina wodier* Roxb., Anacardiaceae)

*Holocacista pariodelta* (Meyrick, 1929): p. 541, comb. n. (*Antispila*)

India, Bihar, Pusa, *Lannea coromandelica* (Houtt.) Merr. (=*Odina wodier* Roxb., Anacardiaceae)

*Holocacista selastis* (Meyrick, 1926): p. 261, comb. n. (*Antispila*)

India, [Karnataka], Karwar, *Psychotria dalzellii* Hook.f. (Rubiaceae)

Unnamed species:

Palearctic species

sp. *Dyerophytum_*UAE

United Arab Emirates, Fujairah, *Dyerophytum indicum* (Gibbs ex Wight) Kuntze (Plumbaginaceae)

African species [see also Appendix A]

sp. *Rhoicissus_tridentata*

South Africa, Rhoicissus tridentata (L. f.) Wild & R. B. Drumm. subsp. cuneifolia (Eckl. & Zeyh.) Urton, *Rhoicissus tomentosa* (Lam.) Wild & R.B.Drumm. (Vitaceae)

sp. *Rhoicissus_tomentosa*

South Africa, *Rhoicissus tomentosa* (Lam.) Wild & R.B.Drumm. (Vitaceae)

sp. *Rhoicissus_*PundaMilia

South Africa, *Rhoicissus digitata* (L.f.) Gilg. & M.Brandt (Vitaceae)

sp. *Cissus_integrifolia*

South Africa, *Cissus integrifolia* (Baker) Planch. (Vitaceae)

sp. *Lannea_*SA

South Africa, *Lannea discolor* (Sond.) Engl. (Anacardiaceae)

sp. *Terminalia_*SA [placement tentative]

South Africa, *Terminalia prunioides* M.A. Lawson (Combretaceae)

Asian species

sp. *Leea_*Borneo

Indonesia, Kalimantan Timur, *Leea indica* (Burm.f.) Merr. (Vitaceae)

sp. *Impatiens_*Vietnam

Vietnam, Cuc Phuong NP, *Impatiens clavigera* Hook. f. (Balsaminaceae)

sp. *Lasianthus_*Borneo

Indonesia, Kalimantan Timur, *Lasianthus* Jack sp. (Rubiaceae)

sp. *Lasianthus_*Sabah

Malaysia, Sabah, *Lasianthus* Jack sp. (Rubiaceae)

sp. *Paedaeria_*Taiwan

Taiwan, *Paederia foetida* L. (Rubiaceae)

Australian species

sp. *Psychotria_*Australia

Australia, Queensland, *Psychotria simmondsiana* F.M.Bailey (Rubiaceae)

sp. *Morinda_*Australia

Australia, Queensland, *Morinda jasminoides* A.Cunn. (Rubiaceae)

sp. *Hibbertia_*Australia

Australia, West Australia, *Hibbertia* Andrews (Dilleniaceae)

### Holocacista rivillei

Taxon classificationAnimaliaLepidopteraHeliozelidae

(Stainton)

Figs 9 , 21 , 23 , 24 , 26 , 29 , 87 , 88 , 105

Elachista rivillei Stainton, 1855: 89.
Holocacista rivillei ; van Nieukerken et al. (2012b): 62 [redescription]; Cean (2014): 385 [record Rumania, description]

#### Note.

**Figures 21–28.**
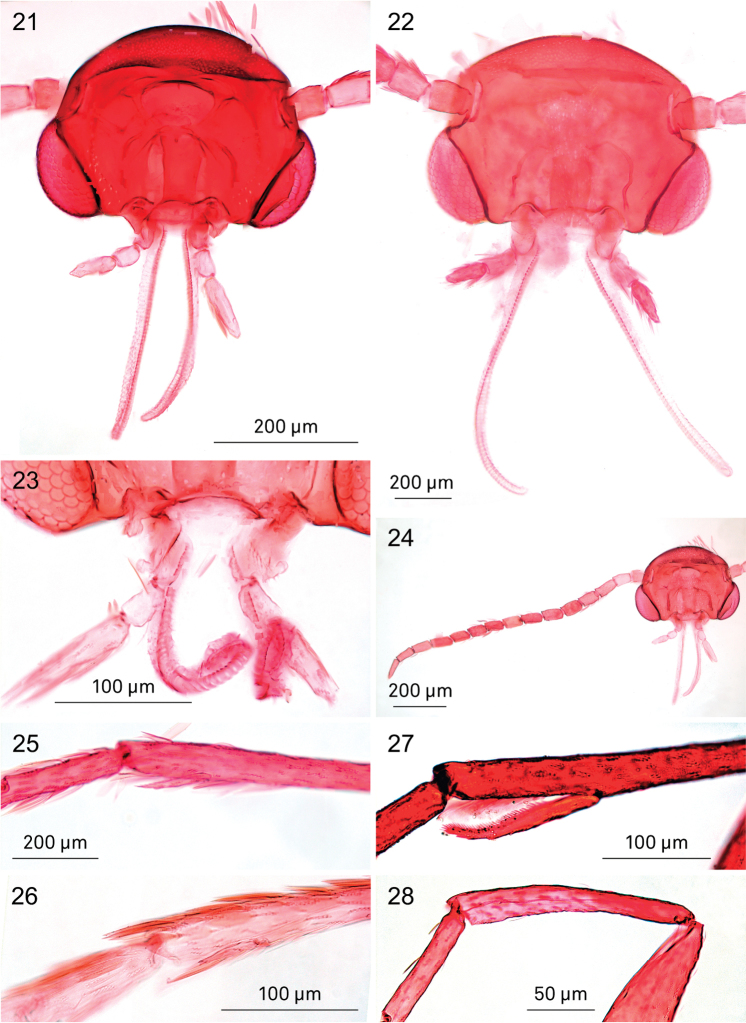
Heliozelidae species, details of adult morphology. **21** *Holocacista rivillei*, male, denuded head, RMNH.INS.24300 **22** *Holocacista capensis*, male, denuded head, RMNH.INS.24445 **23** *Holocacista rivillei*, male, detail mouthparts, RMNH.INS.24443 **24** *Holocacista rivillei*, male, head and antenna, showing 15 segments, RMNH.INS.24300 **25–28** Male foretibia with or without epiphysis: **25** *Holocacista capensis*, small epiphysis, RMNH.INS.24445 **26** *Holocacista rivillei*, small epiphysis, RMNH.INS.24443 **27** *Heliozela sericiella*, large epiphysis, RMNH.INS.24451 **28** *Antispilina ludwigi*, epiphysis absent, RMNH.INS.24448.

**Figures 29, 30.**
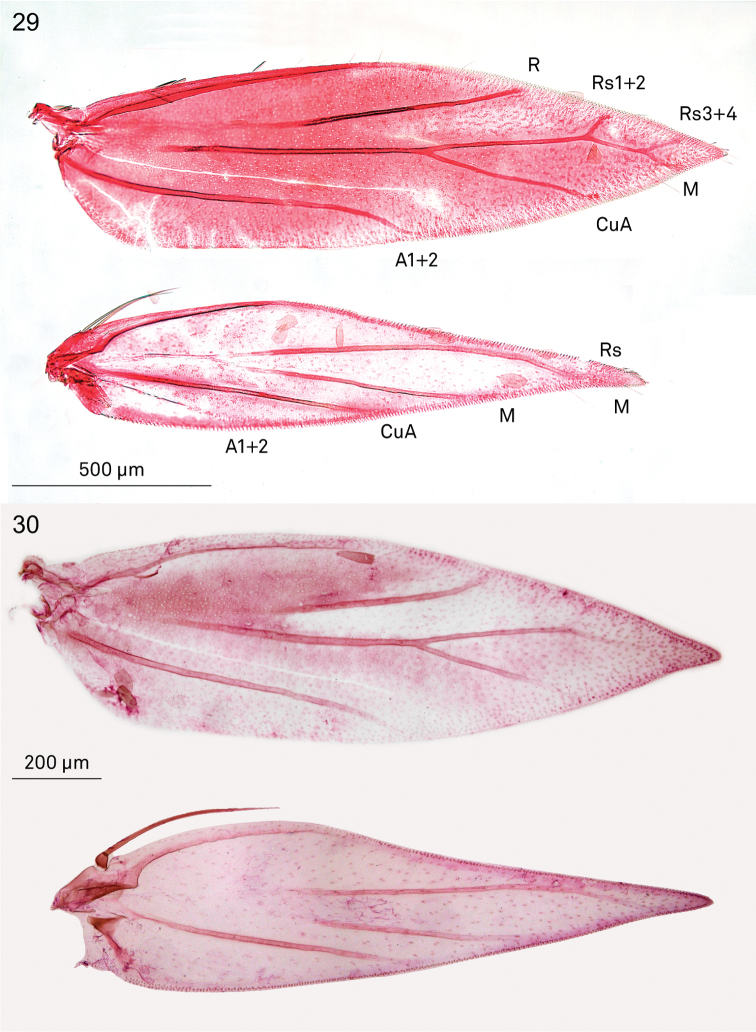
*Holocacista* species, wing venation. **29** *Holocacista rivillei*, female, veins labelled, RMNH.INS.24259 **30** *Holocacista capensis*, male, RMNH.INS.24260.

The type species of *Holocacista* has recently been diagnosed in the context of a study of European (and North American) Vitaceae miners (van Nieukerken et al. 2012b), when the genus was still considered monotypic. Here we briefly diagnose it against other species in the genus, without a full redescription. It should be noted that only material from Italy and Bulgaria has been examined in detail. Morphological details (Figs 21, 23, 24, 26) and venation (Fig. 29) are described under the generic treatment. For a full synonymy we refer to van Nieukerken et al. (2012b).

#### Differential diagnosis

(Fig. 9). Wingspan 4.0–4.5 mm. Antenna ringed, 15 segments; head and thorax bronze grey. Forewing fuscous to black, with golden silvery pattern consisting of four spots, costals distal to dorsals, the first costal and dorsal sometimes united as oblique fascia; a distinct fringe line, fringe silvery white. Differs from South African *Holocacista capensis* and *Holocacista salutans* by more golden shining spots and distinct first costal spot (almost absent or reduced in other species); from *Holocacista varii* and other species by distinct fringe line and ringed antenna.

Male genitalia (Fig. 105, and Figs 48–50 in van Nieukerken et al 2012b). Total length vinculum + tegumen 630–720 μm, phallus 575–630 μm. Pecten with 8–10 teeth. Juxta more elaborate than in South African Vitaceae miners, deeply bifurcate. Phallus without spines on phallocrypt, wrinkled. Female genitalia illustrated by van Nieukerken et al (2012b, Figs 51–52) and Cean (2014).

#### Biology.

**Host plants.** Vitaceae: *Vitis vinifera*, wild and cultivated, possibly also on cultivated *Parthenocissus* Planch. (new record from Russia, Kalmykiya).

**Leafmines** (Figs 87, 88). The egg is inserted usually close to a major vein, probably on leaf underside. The mine is first a gallery, turning from once to several times around the oviposition site and then extends, often along a vein as a rather straight linear mine, occasionally as a serpentine mine; distally enlarging into a small blotch. The frass is black forming a broken line, often not exactly in the middle of the mine; in thicker leaves it may be wider; in the blotch the frass is dispersed; the larva cuts out a case of about 3.3–4 mm × 2.0–2.5 mm.

#### Distribution.

Widespread in southern Europe, Turkey and Central Asia (van Nieukerken et al. 2012b), now also recorded in Romania (Cean 2014). Probably only native in eastern part of its current distribution area.

#### Material examined.

Adults and leafmines: **Bulgaria**: 1♂, 7 adults [sex not determined], Sliven, 5.iv.1928, P. Tschorbadjiev, Genitalia slide JCK7867 (coll. Natural History Museum Sofia). **Italy**: 28 adults (4♂, 1♀ dissected), Vicenza, Borghetto, experimental vineyard, leafmines on *Vitis vinifera*, 2007, emerged i–ii.2009, M. Baldessari (RMNH); 1♂ (dissected), 7♀, many leafmines, larvae, ibidem, 19.viii.2013, EvN2013904, emerged 11.ix–3.x.2013, M. Baldessari (RMNH). **Russia**: leafmines only, Kalmykiya, Elista, Citypark, 26.ix.2000, leafmines on *Parthenocissus*, V. Zolotuhin (coll. Zolotuhin).

### Holocacista capensis sp. n.

Taxon classificationAnimaliaLepidopteraHeliozelidae

http://zoobank.org/1455B935-A7E5-4247-8BAB-47825BAF3048

Figs 1–3 , 22 , 25 , 30 , 37 –53 , 70 –82 , 94–96 , 107–110 , 115 , 116

Antispila sp. Kroon (1999): 83, 120 [on *Vitis* sp.].

#### Type material.

**Holotype male**, South Africa (Western Cape), Paarl NW, De Heuvel estate, 180 m, 16.i.2013, leafmines on *Vitis vinifera* cv ‘Regal’, EvN2013004, emerged 27.i.2013, E.J. van Nieukerken & H. Geertsema, Genitalia slide EvN4622, DNA extracted (RMNH.INS.24622) (RMNH).

#### Differential diagnosis.

Externally *Holocacista capensis* is almost inseparable from other South African Vitaceae-feeding *Holocacista* species, including *Holocacista salutans*. Absence or reduction of the first costal spot in the male, however, may be an indication that the specimen might be *Holocacista capensis*; only study of genitalia allows a firm identification. For differences with *Holocacista varii*, see there. The only South African Vitaceae-feeding “real” *Antispila* species is much larger and has more antennal segments (ca. 26). In male genitalia the configuration of the small number of spines on phallocrypt in combination with the ventrally curved phallus appendix is characteristic, otherwise very similar to *Holocacista salutans* and some of the unnamed *Rhoicissus* miners. Leafmines characterised by the very contorted first part of the mine, which is straighter or shorter in the other species; currently the only known leafminer on *Vitis* in South Africa.

#### Description.

*Male* (Figs 1, 3). Head face and vertex covered with appressed, metallic, silvery-white scales, more brownish grey on vertex. Palpi porrect, white; base of proboscis covered with white scales. Antenna with 16 segments, ringed, each flagellomere with a basal fuscous scale ring and apical white scale ring on upper side, scales on underside all white. Legs grey, tarsi mostly yellowish white, especially on underside. Thorax and forewings ground colour grey brown, slightly irrorate, caused by scales being dark tipped and paler at base. A silver-white pattern on forewing consists of a triangular dorsal spot at 1/4, usually associated with a minor spot of just a few scales at costa, that may be joined to dorsal spot, or even completely absent; a second triangular dorsal spot at 1/2, reaching almost to middle of wing; a triangular costal spot just beyond middle, always separate; fringe line very distinct, demarcated by dark-tipped scales. Terminal fringe silvery white. Hindwings pale grey. Underside of wings fuscous, with white spots visible. Abdomen lead coloured, including vestiture on external genitalia.

*Female* (Fig. 2). Antenna with 16 segments. Colour pattern distinct from male: scales almost uniformly dark fuscous with purplish tinge, resulting in darker, velvety wing colour and contrasting silvery-white pattern; first costal and dorsal spots joined to form a narrow fascia, wider at dorsum; second dorsal and costal spots as in male; fringe line distinct, scales forming cilia line with slightly paler bases. Abdomen almost black, narrowly pointed posteriorly.

*Measurements*. Male: forewing length 1.8–2.3 mm (2.0 ± 0.1, 20) (1 dwarf of 1.55 mm forewing length excluded), wingspan: 3.9–4.9 mm. Female: forewing length 1.9–2.1 mm (2.0 ± 0.1, 14), wingspan 4.0–4.6 mm.

*Male genitalia* (Figs 37–49, 94–96, 107–110, 115). Total length vinculum + tegumen 425–625 µm. Vinculum (S IX) long, reaching anterior margin of segment VI. Tegumen (Figs 41, 46, 110) well sclerotised, with medial, slightly-bilobed posterior projection, one sensilla on each lobe; tegumen dorsally with groups of microtrichia, and two lateral lobes with setae or sensilla; a poorly-sclerotised structure below tegumen may be a reduced uncus. Valva (Figs 40, 43, 108) narrow, apex blunt, with stalked pectinifer halfway to inner margin, pecten comprising 8–11 blunt sensilla, usually same number on both valvae, but sometimes a difference of one. Valva length (without transtilla) 200–230 µm. Transtilla (Figs 41, 109) with long sublateral processes and medial spatulate posterior process, with rounded corners. Juxta elongate, as a narrow ventral process of phallus, attached on phallus near phallocrypt spines (Fig. 48). Phallus (Figs 39, 42, 44, 45, 48, 49, 94–96, 107) long and narrow, ca. 340–425 µm long. Phallocrypt (manica) with some strongly-sclerotised conical spines, arranged asymmetrically; in lateral view (Figs 44, 94–96) one dorsally, curved ventrad, a similar strong one ventrally curved dorsad, latter with 3–4 smaller spines in a row anteriorly; in ventrally mounted specimens spines appear mostly on right side, where phallus is constricted. Phallus outer tube with ventrally-curved appendix ca.103–150 µm long (measured along curve).

*Female genitalia* (Figs 50–53). Length of anterior apophyses 800–900 μm (n=5), posterior apophyses 880–935 μm (n=5). Oviscapt with 5–6 cusps on either side (Figs 51, 52). Ductus spermathecae with many wide convolutions, spermathecal papilla with circle-shaped sclerotisation (Fig. 50).

#### Biology.

**Host plants.** Vitaceae: *Rhoicissus digitata* (L.f.) Gilg. & M. Brandt and various South African grown cultivars of *Vitis vinifera* (e.g., Chardonnay, Chenin Blanc, Red Globe, Régal).

**Leafmines** (Figs 70–75, 79). The egg is inserted on the leaf underside, usually within 1–2 mm from a vein, rarely slightly farther. Freshly expanded foliage is preferentially selected for oviposition, but as egg laying proceeds from early spring to late autumn, it also oviposits on older leaves, even those showing previous feeding. The majority of the mines on *Vitis* (75% of 160 mines from six samples) start at the leaf edge, but even there the egg is always near the vein in the tip of a lobe; some mines originate close to the leaf midrib. Also, the few studied mines on *Rhoicissus* start at the leaf(let) tip. The mine starts as a much contorted narrow gallery, often first in a zigzag pattern with U-turns, eventually enlarging into an irregular wide gallery or a blotch. The frass is brown in the early mine, later black, in a rather thin line in the centre of the gallery; later the frass is in clumps in a wider central line. The whole mine occupies a small area of ca. 12–15 mm long, of which the size depends on leaf thickness; in thin leaves mines are appreciably longer and wider. Mines are very often clustered in groups of 3–5 or even more. The larva cuts out an elliptic case of about 2.5–4.1 mm (3.4 ± 0.3, n=34) × 1.5–3.1 mm (2.3 ± 0.3, n=34) mm wide, ratio 1.2–1.8 (1.5 ± 0.1).

#### Voltinism and habits.

The moth is multivoltine; the first adults appear during early spring (September to October) and a single generation lasts from three to four weeks; peak numbers are reached during February and March at the height of the grape picking season. Moths are still present in April; the last were seen early May; many cocoons overwinter in leaf litter, dropping to the ground and pupating amongst leaf litter or attached to stems and trellises from April onwards, and yielding moths from September onwards. Larvae are present almost continuously from November to early May when the leaves start to wither and drop. Larvae have only once been collected on *Rhoicissus*, these in March. When fully grown, larvae descend from the mines to attach their cocoons upon landing on a variety of objects such as other leaves, berries of grape bunches, trellises or on the bark of the vine itself (Fig. 76).

Moths aggregate and mate in the heat of the day (1100–1400 hrs) on exposed vine foliage, but prefer to oviposit in the shaded canopy conditions under which table grapes are grown; wine grapes, grown in an open cultivation system and fully exposed to the sun are rarely, or at least less seriously attacked.

#### Distribution

(Fig. 115). On native *Rhoicissus* as yet only found once: South Africa, Western Cape (Wilderness). On cultivated *Vitis* from South Africa: Western Cape, Northern Cape and Gauteng.

#### DNA barcode.

We barcoded eight specimens, including the Holotype. All barcodes belong to Barcode Identification Number (BIN):ACG9027, the largest intraspecific distance is 1.4%, between one specimen collected in Gauteng and the rest, collected in the Western Cape.

DNA-Barcode of Holotype, HELA103-14 (658 basepairs):

AACTTTATATTTTATTTTTGGTATTTGAGCGGGATTAGTAGGAACATCAATAAGTTTATTAATTCGTGCTGAATTAGGAATCCCTGGGTCCTTAATTTCTAATGATCAAATTTATAATACTATTGTTACAGCTCATGCATTTATTATAATTTTTTTTATAGTTATACCTATTATAATTGGAGGATTTGGAAATTGATTAGTTCCGTTAATATTAGGAGCCCCAGATATAGCATTTCCTCGTCTTAATAATATAAGTTTTTGACTCCTTCCCCCATCTTTAACATTATTAATTTCAAGAAGATTAGTTGAAATGGGATCAGGAACTGGATGAACTGTCTATCCACCTTTATCTTCCAATATTGCCCATATGGGAACTTCTGTGGATTTAACTATTTTTTCTTTACATTTGGCTGGAATTTCATCTATTTTAGGAGCTGTAAATTTTATTACAACAATTATTAATATAAAACCAGTTAGAATAATATATAATCAACTTTCTTTATTTGTTTGATCTGTGGGTATTACAGCTTTATTACTATTATTATCTTTACCTGTATTAGCTGGAGCTATTACTATATTATTAACTGATCGAAATTTAAATACTTCTTTTTTTGACCCTATGGGAGGAGGAGACCCTATTCTATATCAACATTTATTT

#### Remarks.

The only wild *Rhoicissus* on which mines of *Holocacista capensis* were collected, was identified by Vári in his notebook as *Rhoicissus revoilii*. The single leaf we studied could belong to this species or to *Rhoicissus digitata*, which is very similar. On the basis of the distribution (Palgrave and Palgrave 2002), we conclude that the latter is the most likely, since *Rhoicissus revoilii* is not known to occur in the Western Cape.

Several reared adults were used in 2013 for a rearing experiment on potted plants of *Rhoicissus rhomboidea* (E.Mey. ex Harv.) Planch., bought in the Netherlands. Although the adults lived for several days, no traces of mines were found. Either the species is unsuitable as a hostplant, or these potted plants contained remnants of insecticides. Later, we were more successful with rearing larval offspring from *Vitis*-grown adults from Wellington on potted *Rhoicissus digitata* in the laboratory in Stellenbosch (for resulting leafmines see Fig. 79). The main aim of this preliminary study was to detect whether moths reared on *Vitis vinifera* would readily breed on (caged) *Rhoicissus digitata*; moths emerging from grapevine leaf litter or sampled foliage were released into the caged *Rhoicissus*. The latter was readily infested, often resulting in the entire leaf being consumed by the larvae.

Other live cocoons were sent in 2013 to Lund, Sweden, emerged there, and have been used for pheromone studies (Wang et al. 2015).

#### Material examined.

Adults and leafmines: **South Africa, Gauteng**: 3♂, Pretoria, Roodeplaat, 1245 m, leafmines *Vitis vinifera*, emerged 10–12.x.1990, S. Marais, Genitalia slide EvN4264, DNA extracted (RMNH.INS.24264) (HG, RMNH); 3♂, 5♀, same locality, emerged 4–14.iv.2012, D. Visser (HG, RMNH); 2♀ [5 more specimens in TMSA], Pretoria, emerged 2–6.xi.1950, L. Vári, Genitalia slide TM6830, Wing slide TM 2414 (TMSA); 1♀ [1 more specimen in TMSA], 1 herbarium sheet with 6 leafmines on 4 leaves, Pretoria, 8.iii.1953, Ac. no. 660, leafmines on *Vitis vinifera*, emerged 10–31.iii.1953, L. Vári (TMSA); 1♂, 1♀, 1 herbarium sheet with ca. 13 leafmines in 4 leaves, Pretoria, in own garden, 21.x.1953, Ac. no. 866, leafmines on *Vitis vinifera*, emerged 26.x–3.xi.1953, L. Vári (TMSA); 1♂, larvae and leafmines, Roodeplaat exp. Farm, 1168 m, 23.i.2013, leafmines *Vitis vinifera*, EvN2013025–026, E.J. van Nieukerken & S. Richter, 1 larva DNA extracted (RMNH.INS.29586). **Northern Cape**: 9♂, 14♀ [unmounted], Vaalhartz Research Stn., Jan Kempdorp near Kimberly, 27.ii.1980 [emergence date?], W. v.d. Westhuyzen (TMSA). **Western Cape**: 2♂, 3♀, Cape Town, Woodstock, cocoons collected on *Vitis vinifera*, 26.ii.2012, emerged 5–13.iii.2012, M. Wohlfarter; 1♂, Oudtshoorn, March 1998, on urban vine, H. Geertsema (HG); 81♂, 58♀, Paarl, nr Windmeul, 168 m, leafmines/cocoons on *Vitis vinifera*, emerged 1.ii–30.iii.2012, H. Geertsema, Genitalia slides EvN4260♂, 4261♀, 4262♂, 4263♀; complete adults on slide EvN4445♂, 4446♂, 4447♂, DNA extracted (RMNH.INS.24260, 24261, 24262, 24263, 24445, 24446, 24447) (HG, RMNH); 3♂, 2♀, Paarl NW, Nelson estate, 125 m, 15.i.2013, leafmines on *Vitis vinifera* cv ‘Chenin Blanc’, EvN2013002, emerged 18.i–1.ii.2013, E.J. van Nieukerken & H. Geertsema; 6♂, 10♀, 4 larvae, ibidem, 130 m, leafmines on *Vitis vinifera* cv ‘’Chardonnay’, EvN2013003, emerged 24.i–5.ii.2013, Genitalia slide EvN4624♀, DNA extracted (RMNH.INS.24624), larvae RMNH.INS.2956265; 6♂, 11♀, 6 larvae, Paarl NW, De Heuvel estate, 180 m, 16.i.2013, leafmines on *Vitis vinifera* cv ‘Regal’, EvN2013004, emerged 20–27.i.2013, E.J. van Nieukerken & H. Geertsema, larvae RMNH.INS.29578–83; 2♂, 3♀, ibidem, leafmines on *Vitis vinifera* cv ‘Red globe’, EvN2013005, emerged 26.i–4.ii.2013; 1♂, 3♀, ibidem, 25.i.2013, leafmines on *Vitis vinifera* cv ‘Red globe’, EvN2013030, emerged 1–6.ii.2013, E.J. van Nieukerken & H. Geertsema (RMNH, HG); 5 adults, Somerset, 23.ii.2012, cocoons collected on *Vitis vinifera*, emerged 27.ii–5.iii.2012, O. Lotter; 1♀, Wellington, emerged 25.xii.2014, leafmines on *Vitis vinifera*, L. Torrance (HG). 3♂, 1♀ [8 more specimens in TMSA], 1 leaf with 6 mines, Wilderness, Kaaimans River, 15.iii.1954, Ac. no. 1093, leafmines on *Rhoicissus digitata* [in notebook Vári as *Rhoicissus revoilii*], emerged 4–5.iv.1954, L. Vári, Genitalia slide EvN4381♂, DNA extracted (RMNH.INS.24381) (TMSA).

#### Additional data

[leafmines and larvae collected, no adults kept in collection]. **South Africa, Western Cape**: 11♂, 21♀ [reared from 50 cocoons in Lund, Sweden and used for pheromone studies], Paarl NW, De Heuvel estate, 180 m, 25.i.2013, leafmines on *Vitis vinifera* cv ‘Regal’, EvN2013029, emerged 2–15.ii.2013, E.J. van Nieukerken & H. Geertsema; several adults, Wellington, emerged xii.2014, ex *Vitis vinifera* laboratory bred on *Rhoicissus digitata*, L. Torrance (HG).

### Holocacista salutans

Taxon classificationAnimaliaLepidopteraHeliozelidae

(Meyrick) comb. n.

Figs 4 , 54–58 , 65 , 66 , 85 , 86 , 97–100 , 111 , 113 , 115

Antispila salutans Meyrick, 1921: 108. 5 Syntypes ♂♀: South Africa, [KwaZulu Natal], Durban, x.[19]18/19, v.d. Merwe (TMSA, BMNH) [partly examined].
Antispila salutans ; Vári and Kroon (1986): 154; Vári et al. (2002): 10; De Prins and De Prins (2014): database.

#### Differential diagnosis.

Externally *Holocacista salutans* hardly differs from *Holocacista capensis*, but the male usually has a costal spot at 1/3 from base, albeit very small. The only consistent characters to separate it from *Holocacista capensis* are in the male genitalia: the row of larger spines dorsally on the phallocrypt, whereas *Holocacista capensis* has a row ventrally and just a single spine ventrally; also the shape of the transtilla *Holocacista salutans* differs from that in *capensis*. The leafmines of *Holocacista salutans* have the gallery mine with wider frass, more clumped and not zigzag as in *Holocacista capensis*.

#### Description.

*Male* (Fig. 4). Head: face and vertex covered with appressed, metallic, silvery-white scales, more brownish grey on vertex. Palpi porrect, white; base of proboscis covered with white scales. Antenna with 16 segments, ringed, each flagellomere with a basal fuscous scale ring and apical white scale ring on upper side, scales on underside all white. Legs grey, tarsi mostly yellowish white, especially on underside. Thorax and forewings ground colour grey brown, slightly irrorate, caused by scales being dark tipped and paler at base. A silver-white pattern on forewing consists of a triangular dorsal spot at 1/4 from base, a smaller spot at costa, sometimes joined to dorsal spot as a narrow fascia; a second triangular dorsal spot at 1/2, reaching almost to middle of wing; a triangular costal spot just beyond middle, always separate; fringe line very distinct, demarcated by dark-tipped scales. Terminal fringe silvery white. Hindwings pale grey. Underside of wings fuscous, with white spots visible. Abdomen lead grey, including vestiture on external genitalia.

*Female*. Antenna with 16 segments. Colour pattern distinct from male: scales almost uniformly dark fuscous with purplish tinge, resulting in darker, velvety wing colour and contrasting silvery-white pattern; first costal and dorsal spots always joined to form a narrow fascia, wider at dorsum; second dorsal and costal spots as in male; fringe line distinct, scales forming cilia line with slightly paler bases. Abdomen almost black, narrowly pointed posteriorly.

*Measurements*. Male: forewing length 1.7–2.3 mm (2.0 ± 0.2, 6), wingspan: 4.0–5.0 mm. Female: forewing length ca. 2.0 mm (n=3), wingspan ca. 4.5 mm.

*Male genitalia* (Figs 54–58, 97–100, 111, 113). Total length vinculum + tegumen ca. 460–490 µm (n=3). Vinculum (S IX) long, reaching anterior margin of segment VI. Tegumen and uncus well sclerotised, with two medial projections, probably representing tegumen and uncus, dorsalmost projection very similar to tegumen of *Holocacista capensis*, ventral one truncate, slightly excavated posteriorly, with serrate margins. Valva narrow, apex blunt, with stalked pectinifer halfway along inner margin, pecten comprising 8–10 blunt sensilla. Valva length (without transtilla) ca. 165–215 µm. Transtilla with long sublateral processes and medial spatulate posterior process, with produced lateral corners (Fig. 111). Juxta (Fig. 56) elongate, as a narrow ventral process of phallus, attached to phallus near phallocrypt spines. Phallus (Figs 56, 97–100) long and narrow, ca. 390–430 µm long. Phallocrypt (manica) with two rows of strongly-sclerotised conical spines, arranged symmetrically; in lateral view seen dorsally, all curved ventrad, more than 6–7 spines in a row; a group of small spines posterior to these. Phallus outer tube not constricted, with ventrally-curved appendix of ca. 105–125 µm long (measured along curve).

*Female genitalia* (Figs 65, 66). Length of anterior apophyses 850 μm (n=1), posterior apophyses 890 μm (n=1). Oviscapt not yet studied in ventral view. Ductus spermathecae with many wide convolutions, spermathecal papilla with circle-shaped sclerotisation (Fig. 66).

#### Biology.

**Host plants.** Vitaceae: *Rhoicissus digitata* (L.f.) Gilg. & M. Brandt, *Rhoicissus revoilii* Planch., *Rhoicissus tomentosa* (Lam.) Wild. & R.B. Drumm. and *Cissus cornifolia* (Baker) Planch. Records from Rhoicissus tridentata (L. f.) Wild. & R. B. Drumm. subsp. cuneifolia (Eckl. & Zeyh.) Urton require confirmation (see below).

**Leafmines** (Figs 85, 86). The egg is inserted on the leaf underside, usually close to a vein; some mines start at the leaf edge. The mine starts as a much contorted narrow gallery with all convolutions close to each other, hardly leaving leaf tissue between them. Later, the mine enlarging into an irregular wide gallery or a blotch. The frass is black throughout, clumped and almost filling the gallery, but with space between the clumps. Mines are very often clustered in groups. The larva cuts out an elliptic case of about 3 mm long and 2 mm wide.

**Voltinism.** Larvae have been found from March to June, in September and again from December to January; adults usually emerge between 3–8 weeks after collecting of leafmines; probably multiple overlapping generations.

#### Distribution

(Fig. 115). South Africa: KwaZulu-Natal, Limpopo and Zimbabwe: Masvingo. Records from Gauteng (Pretoria) need confirmation, several leafmines from *Rhoicissus tridentata* resemble those of *Holocacista salutans* on other hosts, but we have yet no proof from adults that they are this species.

#### Remarks.

Meyrick (1921) described *Antispila salutans* from five specimens from “Natal, Durban, in October (Janse).” They were part of a much larger series, of which the labels in the Ditsong Museum (former Transvaal Museum) give more information: all from Durban, collected by v.d. Merwe, Coll. Janse. Some are dated 10.10.18 [in hand], others x.19 or xi.19 [in print], those dated x.19 also have a label with the text “Ac. n. 453” [Accession number 453]. Many specimens have a cocoon added, showing that they have been reared. The five specimens in London are merely labelled (in Meyrick’s hand) “Durban, Natal, AJTJ, 10 [or 11].19”. Meyrick usually replaced original labels of specimens that he kept for his own collection with shorter ones in his own hand or print (Clarke 1955). Meyrick (1921) wrote in the introduction of his paper: “The types of those new species received from Mr A. J. T. Janse are contained in his collection, ….” Three specimens of *Antispila salutans* are placed in the type collection in Pretoria under the type numbers 109–111. In addition to the locality label, they have a type label in red ink, with name and type number, and an additional label, printed in black, with a 4-digit number, split in two rows. Such labels are always attached to Janse specimens studied by Meyrick, but no registry book is available in Pretoria with more information (Martin Krüger, personal communication). So if we regard these as real syntypes, even though one of these was labelled with ‘xi’, thus from November, only two of the specimens in London can be regarded as the remaining syntypes, and all other specimens are just topotypical, but not types (see also Razowski and Krüger 2007). For the time being we cannot make a decision as to which specimens in London are actual syntypes, and suggest that the male specimen in the type collection in Pretoria with “Type No. 109” is probably the best candidate to be selected as Lectotype, preferably during a full revision. For now a Lectotype selection does not seem necessary.

Unfortunately, we have not been able to find information on the rearing and hostplant of van der Merwe’s series. The Accession number 453 on some labels had previously been misinterpreted as a number of Vári, who labelled all his reared material with such numbers [probably following up on Janse’s system, but with new numbers]. The leaf with mines that was pinned in the *salutans* box belongs to *Bridelia cathartica* Bert. (Euphorbiaceae), has probable Coleoptera mines, and has Vári’s number 453. Obviously, this has nothing to do with the heliozelid. There are no notebooks of Janse left that could shed light on this number (Martin Krüger, personal communication).

Unfortunately, we did not find recent material of this species and are therefore as yet unable to give the DNA barcode.

#### Material examined.

**Syntypes**. **South Africa, KwaZulu-Natal**: 1♂, “DURBAN / 10.10 / v.d. Merwe 18/ Coll. Janse” [black print, date in hand, black cadre]; “24 / 93” [black print]; “Antispila /salutans /Type No. 109”[hand, red ink, “Type No” in print]. 1♂, “DURBAN / 10.10 / v.d. Merwe 18/ Coll. Janse” [black print, date in hand, black cadre]; “24 / 93” [black print];”Antispila /salutans /Cotype No. 111” [hand, red ink, “Cotype No” in print]. 1 adult, “DURBAN / v.d. Merwe xi.19/ Coll. Janse” [black print, black cadre]; “29/ 28” [black print]; “Antispila /salutans /Cotype No. 110” [hand, red ink, “Cotype No” in print]. 4 specimens including 2 possible Syntypes in London, not examined.

#### Non-type material.

Adults and leafmines: **South Africa, Kwazulu-Natal**, 2♂, 1♀, Durban, emerged x and xi.1919, van der Merwe [ex coll. Janse], genitalia slide TM4023 (♂), wing slide TM1585 (♀); 5♂, 3♀, 1 leaf with 8 mines, Jozini Dam [Pongolapoortdam], Lebombo Mts., 14.i.1965, Ac. no 2788, leafmines on *Rhoicissus tomentosa*, emerged 27.i–5.ii1965, L. Vári, genitalia slides EvN4384 (♂),EvN4668 (♀); 1♂, 2♀, Umhlanga Rocks, 9–16.vi.1968, Ac. no 2944, leafmines on *Rhoicissus revoilii*, emerged 2–5.viii.1968, L. Vári; 1♂, 1♀, Umhlanga Rocks, 25.iii.1975, Ac. no 3342, leafmines on *Rhoicissus revoilii*, emerged 10–11.iv.1975, L. Vári, genitalia slide EvN4383 (♂); **Limpopo**: 1♂, 1♀, 1 leaf with 3 mines, Cyprus Farm, nr. Ofcolaco, 20.ix.1960, Ac. no 2247, leafmines on *Rhoicissus tomentosa*, emerged 11–13.x.1960, L. Vári; 6 leafmines on 6 leaves, Debengeni, De Hoek, Waterfalls, 15.vi.1954, Ac. no. 1329, leafmines on *Rhoicissus revoilii*, L. Vári; 1♂, 5 mines on 2 leaves, Louis Trichardt, 17.iii.1964, Ac. no 2693, leafmines on *Rhoicissus tomentosa*, emerged 31.iii–5.iv.1964, L. Vári. **Zimbabwe, Masvingo**: 2♂, 11 mines on 11 leaves, Lundi, 22.iv.1956, Ac. no 1916, leafmines on *Cissus cornifolia*, emerged 1–30.vi.1956, L. Vári; genitalia slide EvN4386 (♂) (all TMSA).

### Holocacista varii

Taxon classificationAnimaliaLepidopteraHeliozelidae

(Mey, 2011) comb. n.

Figs 5 , 6 , 59–64 , 67–69 , 89 , 90 , 104 , 112 , 114 , 116

Antispilina varii Mey, 2011: 156. Holotype ♂ RSA, Cape Town, 26.3.1954, bred from *Pelargonium cucullatum* from slopes of the Table Mtn., Vári Ac. No. 1047, leg. 4.3.1954, L. Vári, genitalia slide Mey (TMSA) [not examined].

#### Differential diagnosis.

*Holocacista varii* is the only species similar to *Holocacista capensis* that occurs probably commonly in the natural habitats near the grape growing areas of Western Cape and thus could potentially be confused with it. It is distinctly larger, and the forewings are more shining bronze than those of *Holocacista capensis*. Moreover, the male and female have a complete fascia at 1/3 from forewing base that is not narrower at the costa and the antennae are not ringed. In male genitalia, *Holocacista varii* lacks the larger spines on the phallocrypt, and has a more developed juxta; further, the dorsal row of spines on the tegumen is characteristic and the shape of the transtilla differs. The female genitalia have more elaborate sclerotisations, and the apophyses are longer.

#### Redescription.

*Male* (Fig. 5). Head face and vertex covered with appressed, metallic, pale-bronze scales. Palpi porrect, white; base of proboscis covered with white scales. Antenna with ca. 20 segments, uniform bronze brown, scales on underside all white. Legs grey, tarsi mostly yellowish white, especially on underside. Thorax and forewings grey brown with some bronze lustre, with silver-white patterning; an oblique fascia at 1/4, hardly narrower at costa; a slightly triangular dorsal spot at 1/2, not reaching middle of wing; a triangular or squarish costal spot just beyond middle; fringe line not very distinct, demarcating scales not conspicuously dark tipped. Terminal fringe silvery white. Hindwings pale grey. Underside of wings fuscous. Abdomen lead grey, including vestiture on external genitalia.

*Female* (Fig. 6). Antenna with ca. 19 segments. Colour pattern different from male: scales more uniformly bronze brown, with strong lustre, and contrasting silvery-white pattern.

*Measurements*. Male: forewing length 2.4–2.8 mm (2.5 ± 0.1, 6), wingspan: 5.0–5.7 mm. Female: forewing length 2.1–2.6 mm (n=3), wingspan 4.5–5.6 mm.

*Male genitalia* (Figs 59–64, 104, 112, 114). Total length vinculum + tegumen ca. 670 µm. Vinculum (S IX) long, reaching anterior margin of segment VI. Tegumen (Figs 60, 61, 114) well sclerotised, with medial, blunt posterior projection, with several setae; tegumen dorsally with a transverse keel with many strong spines in posterior direction. Valva (Fig. 63) narrow, basally wider, apex blunt, with stalked pectinifer halfway along inner margin, pecten comprising 7 or 8 blunt sensilla. Valva length (without transtilla) ca. 265 µm. Transtilla (Figs 64, 112) with relatively long sublateral processes and medial spatulate posterior process, indented posteriorly. Juxta (Fig. 62) well developed, split into an elongate process ventral to phallus and a furcate process dorsal to phallus. Phallus (Figs 62, 104) long and narrow, ca. 540 µm long. Phallocrypt (manica) slightly spinulose posteriorly, no strong spines present. Phallus in lateral view a distinctly-curved outer tube with ventrally-curved appendix, the latter almost straight, ca. 120 µm long (measured as curve).

*Female genitalia* (Figs 67–69). Length of anterior apophyses ca. 935 μm (n=1), posterior apophyses 1020 μm (n=1). Oviscapt with 4 or 5 cusps at either side (Fig. 69). Ductus spermathecae with many wide convolutions, spermathecal papilla with circle-shaped sclerotisation, other elaborate sclerotisations in vestibulum.

#### Biology.

**Host plants.** Geraniaceae; most commonly found on *Pelargonium cucullatum* (L.) L’Hérit., a common plant in Fynbos of Cape Peninsula and the western part of the Western Cape; single records on *Pelargonium panduriforme* Eckl. & Zeyh., *Pelargonium hispidum* Willd. and *Pelargonium citronellum* J.J.A. Van der Walt.

**Leafmines** (Figs 89–90). The egg is inserted at any place of the leaf underside, usually not far from a vein. The mine starts towards a vein and then follows the vein as a narrow gallery of ca. 2 cm, eventually rather suddenly enlarging into a more or less triangular or elongate blotch, usually after the mine makes a turn of 180°. In thin leaves the blotch can be very elongated. The frass in the early mine is a narrow black line, in the blotch the frass is typically clumped near the entrance. Mines occur either singly or with a few together on one leaf. The larva cuts out an elliptic case of about 3.4–3.7 mm long × 1.6–2.2 mm wide. The larva probably descends with its cocoon into leaf litter before pupation.

**Voltinism.** Larvae are found between mid-September and April, and are apparently absent in winter; adults usually emerge between 3–6 weeks after collecting the larvae, suggesting there are multiple overlapping generations. The specimen from Worcester (October) is the only record of an adult taken in the wild.

#### Distribution

(Fig. 116). South Africa: Western Cape, Eastern Cape (new record). The species is abundant on Table Mountain and in the Cape Peninsula, but it is here also recorded from other Fynbos localities around Stellenbosch and Worcester, and Vári also reared it from Zuurberg Pass in Eastern Cape, so we assume a wide distribution in Western and Eastern Cape.

#### DNA barcode.

We barcoded four specimens (three from Table Mountain, one from Stellenbosch), with a maximum intraspecific distance of 1.55%, within the same population on Table Mountain. The BIN is BOLD:ACG8941.

#### Remarks.

In the original description Mey (2011) mentioned that the valval pecten has 13–15 spines, a number that we cannot confirm from the few specimens studied. He further considered the phallus appendix as a cornutus. This appendix, however, is not attached to the vesica, but is an unmovable extension of the phallic tube. Mey (2011) placed this species in *Antispilina* on the basis of its venation, overlooking the fact that *Holocacista* has the same venation. *Antispilina* is a small Palearctic genus, feeding on Polygonaceae, with some new species in the course of description (B.W. Lee et al. in preparation). The placement of this species in *Holocacista*, which is diverse in Africa, makes much more sense. Possibly the ancestor of *Holocacista varii* shifted hosts from Vitaceae to *Pelargonium*.

Many of the specimens listed under material have only been briefly examined by EvN during his visit to the Ditsong Museum, hence the absence of indication of sex.

#### Material examined.

**Eastern Cape**: 1 adult, Zuurberg Pass, south slopes, 22.iii.1954, 11 mines, Pelargonium sp., 1 adult emerged 20.iv.1954, L. Vári (TMSA). **Western Cape**: 5 adults, Bloubergstrand, 3.x.1974, Ac. no. 3308, leafmines on *Pelargonium*, emerged 26–30.x.1974, L. Vári (TMSA); 2 adults, ibidem, 13.x.1975, Ac. no. 3496, leafmines on *Pelargonium*, emerged 14.xi.1975, L. Vári (TMSA); 6 adults, Cape of Good Hope Nature Reserve, 26.x.1966, Ac. no. 2851, leafmines on *Pelargonium*, emerged 21–23.xi.1966, L. Vári (TMSA); 8 adults, ibidem, Ac. no. 288527.x.1967, emerged 20–22.xi.1967, L. Vári (TMSA); 4 adults, Cape Peninsula, Bakoven, 29.x.1975, Ac. no. 3499, leafmines on *Pelargonium*, emerged 17–19.xi.1975, L. Vári (TMSA); 5 adults, Cape Peninsula, Hout Bay, 11.xi.1954, Ac. no. 1357 leafmines on *Pelargonium cucullatum*, emerged 7–21.xii.1954, L. Vári (TMSA); 2 adults (paratypes), ibidem, 14.ix.1966, Ac. no. 2846, emerged 21.xi.1966, L. Vári (TMSA); 10 adults (paratypes), Cape Peninsula, nr. Muizenberg, Steenberg, 10.xi.1979, Ac. no. 3764, leafmines on *Pelargonium*, emerged 15.xi–10.xii.1979, L. Vári (TMSA); 1♂, 1♀, 1 adult (paratypes), Cape Town, Kirstenbosch, 17.xi.1954, Ac. no. 1365, leafmines on *Pelargonium cucullatum*, emerged 11–14.xii.1954, L. Vári (MHUB, TMSA); 1 adult, ibidem, emerged 5–29.xii.1954, A.J.T. Janse (TMSA); 2 adults (paratypes), ibidem, 14.ix.1962, Ac. no. 2535, emerged 1–17.x.1962, L. Vári (TMSA); 1♂, 1♀, ibidem, 23.xi.2014, leafmines on *Pelargonium citronelium*, emerged 11.xii.2014, L. Torrance & H. Geertsema (USEC); 1♂, 1♀, ibidem, 23.xi.2014, leafmines on *Pelargonium cucullatum*, emerged 11–14.xii.2014, L. Torrance & H. Geertsema (USEC).

6♂, 4♀ (paratypes), 1 adult, Cape Town, slopes Table Mt., 4.iii.1954, Ac. no. 1047, leafmines on *Pelargonium cucullatum*, emerged 26.iii–3.iv.1954, L. Vári (TMSA); 9 adults, Noordhoek, leafmines on *Pelargonium*, pupa 10–14.v.1984, emerged 28–31.v.1984, H. Geertsema (TMSA); leafmines, Stellenbosch, Botanical Garden, 122 m, 27.i.2013, EvN2013032, leafmines on *Pelargonium panduriforme*, E.J. van Nieukerken (RMNH); 1 larva, ibidem, 27.i.2013, EvN2013033, leafmines on *Pelargonium hispidum*, E.J. van Nieukerken (RMNH); 3 larvae, Stellenbosch, Jonkershoek, 390 m, 18.i.2013, EvN2013017, leafmines on *Pelargonium cucullatum*, E.J. van Nieukerken & H. Geertsema (RMNH); 3♂, 4♀, 3 larvae, Table mountain NP, Cecilia, nr Klaasenskop, 385 m, 19.i.2013, EvN2013022, leafmines on *Pelargonium cucullatum*, emerged 11–22.ii.2013, E.J. van Nieukerken (RMNH); 1♂, 1♀, 1 larva, Table mountain NP, Cecilia, parking lot, 180 m, 19.i.2013, EvN2013024, leafmines on *Pelargonium cucullatum*, emerged 24.i–12.ii.2013, E.J. van Nieukerken & H. Geertsema (RMNH); 1♂, Worcester, Fairy Glen, 15–19.x.1966, L. Vári & Potgieter (TMSA).

#### Other material.

Leafmines, observation, Western Cape, Ashton, 2014, L. Torrance & H. Geertsema.

**Figures 31–36.**
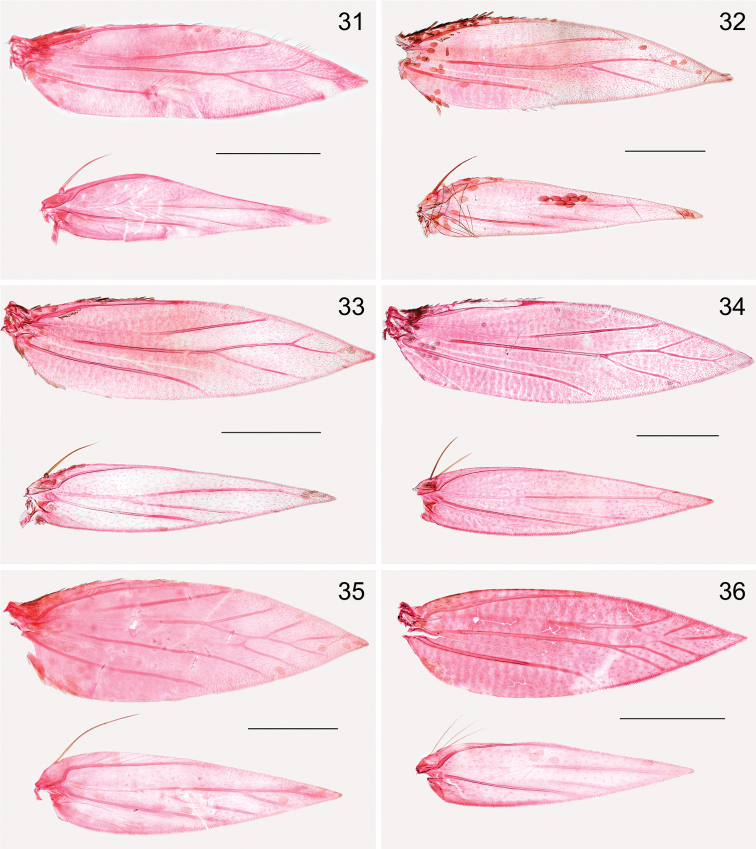
Heliozelidae species, wing venation **31** *Holocacista micrarcha*, male, BM34300 **32** *Holocacista selastis*, male, BM34299 **33** *Holocacista* sp. *Psychotria_*Australia, male, RMNH.INS.24367 **34** *Holocacista* sp. *Impatiens_*Vietnam, female, RMNH.INS.24368 **35** *Antispila argostoma*, male, BM34298 **36** *Heliozela anna*, female, BM34301. Scales 0.5 mm.

**Figures 37–41.**
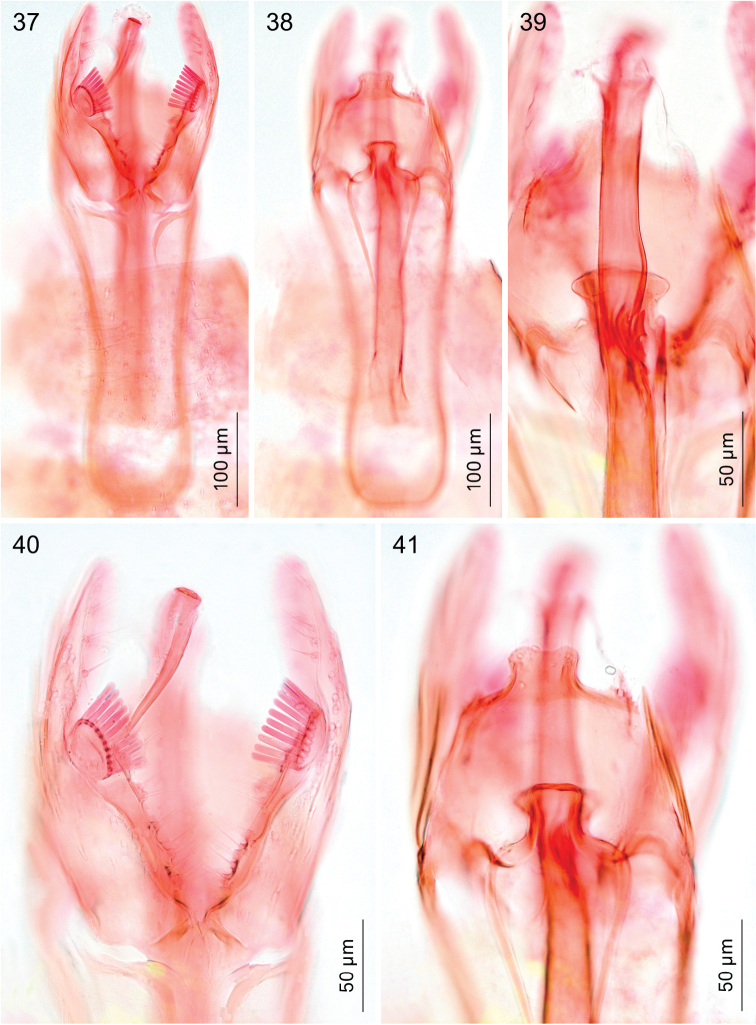
*Holocacista capensis*, male genitalia in ventral view, RMNH.INS.24445; **37** and **40** focussed on ventral side, showing valvae and phallus tip; others more dorsally, showing tegumen and transtilla.

**Figures 42–49.**
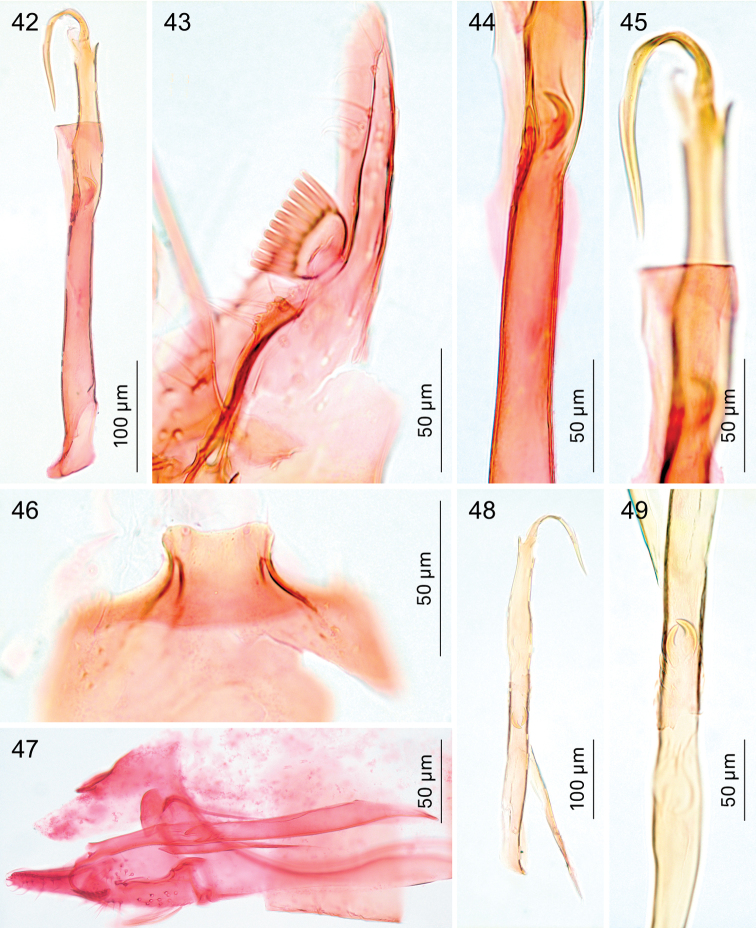
*Holocacista capensis*, male genitalia, details. **42–46** Holotype, RMNH.INS.24642, phallus in ventro-lateral view (42, 44, 45); valva in ventral view (43); tegumen in dorsal view (46) **47** Genitalia in lateral view, RMNH.INS.24446 **48, 49** phallus and juxta, lateral view, slide JCK7813.

**Figures 50–53.**
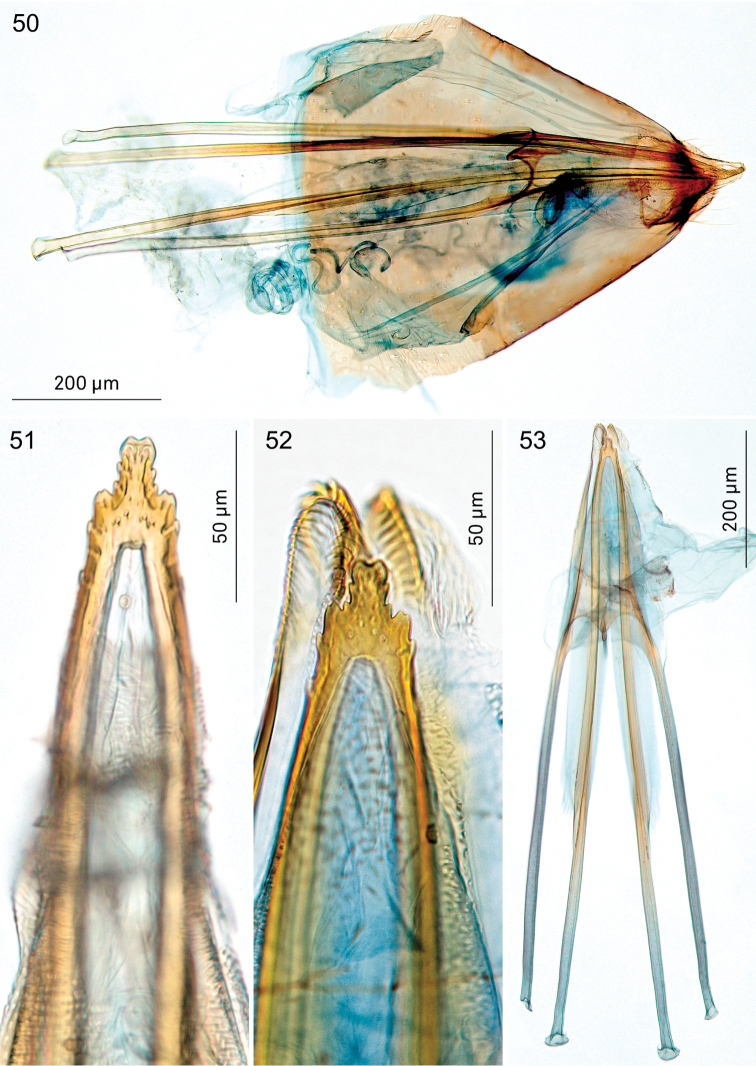
*Holocacista capensis*, female genitalia. **50** lateral view, RMNH.INS.24261 **51–53** Oviscapt detail (**51, 52**) or complete apophyses in ventral view, RMNH.INS.24625 (**51**), RMNH.INS.24624 (**52, 53**).

**Figures 54–58.**
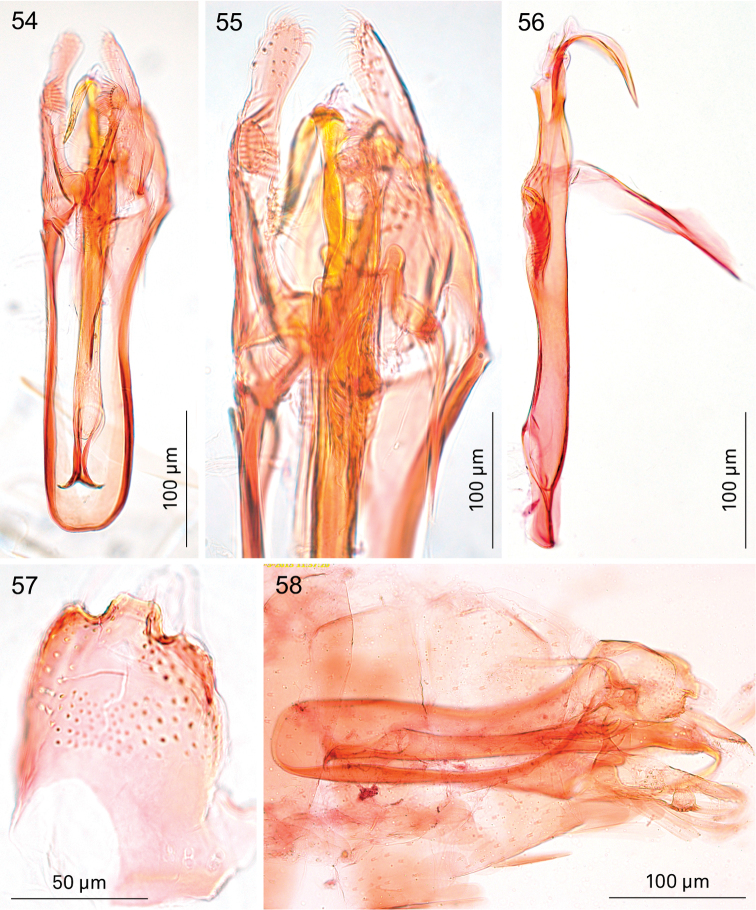
*Holocacista salutans*, male genitalia. **54, 55** ventro-lateral view, Genitalia slide EvN4383 **56** phallus in lateral view, Genitalia slide EvN4384 **57** tegumen in almost dorsal view, Genitalia slide EvN4384 **58** possible syntype in lateral view, Genitalia slide TM4023.

**Figures 59–64.**
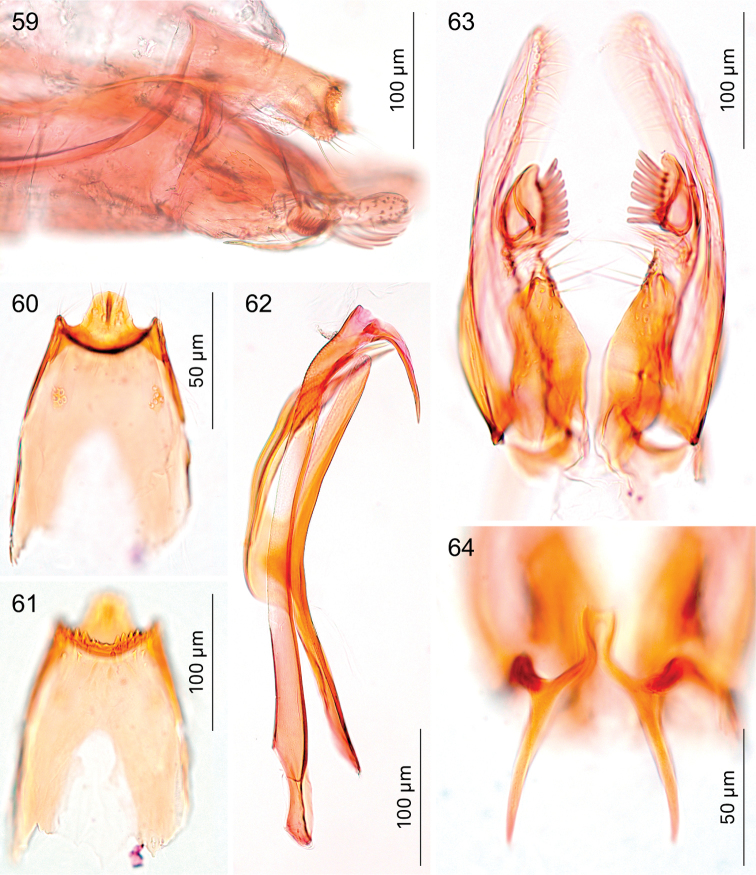
*Holocacista varii*, male genitalia. **59** ventral view, photographed in glycerin, Genitalia slide EvN4388 **60–64** details, RMNH.INS.24623 **60, 61** tegumen, respectively more ventrally and dorsally focussed **62** phallus and juxta in lateral view **63** valvae in ventral view **64** transtilla ventral view, focussed more dorsally.

**Figures 65–69.**
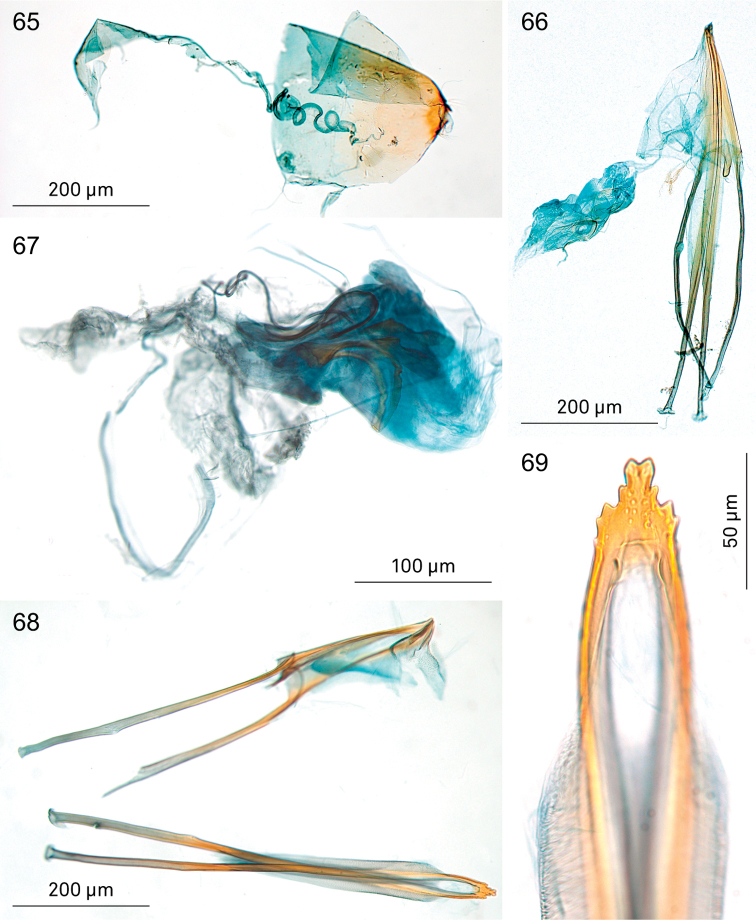
*Holocacista* species, female genitalia. **65, 66** *Holocacista salutans*, lateral view, RMNH.INS.24668 **67–69** *Holocacista varii*, RMNH.INS.24625: **67** internal genitalia in lateral view **68** apophyses in more or less ventral view **69** oviscapt detail, ventral view.

**Figures 70–75.**
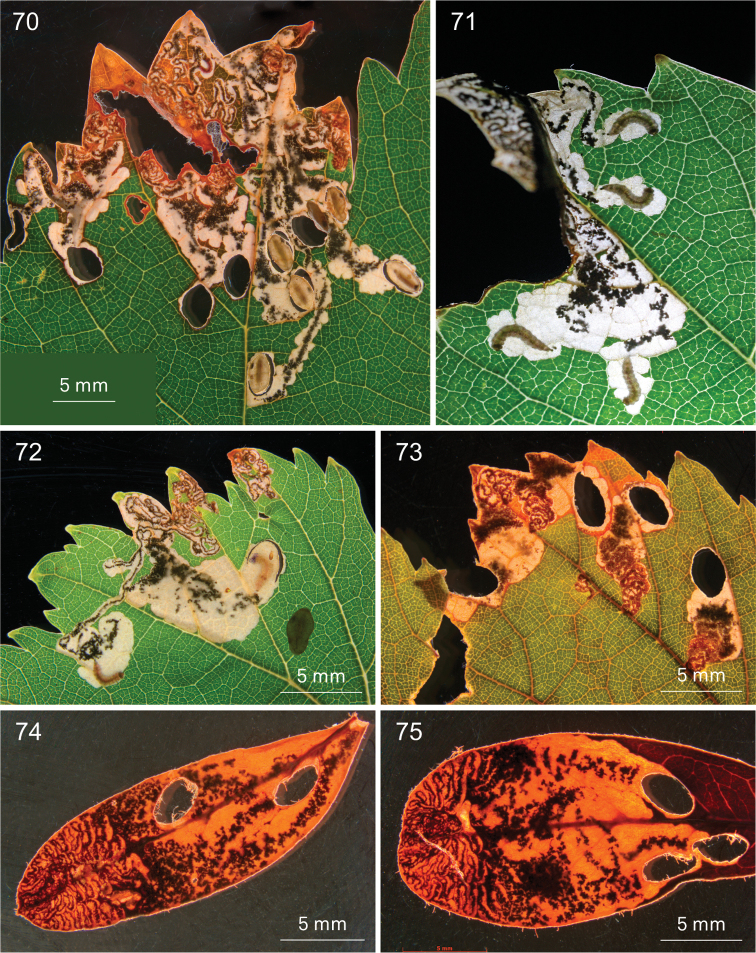
*Holocacista capensis*, leafmines on *Vitis vinifera*, Paarl (70–73) and *Rhoicissus digitata*, Wilderness (74, 75) **70** EvN2013029, 25 January 2013 **71** 16 January 2013 **72** EvN2013029, 25 January 2013 **73** 2013003, 15 January 2013 **74, 75** Vári Ac. No. 1093, 15.iii.1954 (dried leafmines).

**Figures 76–82.**
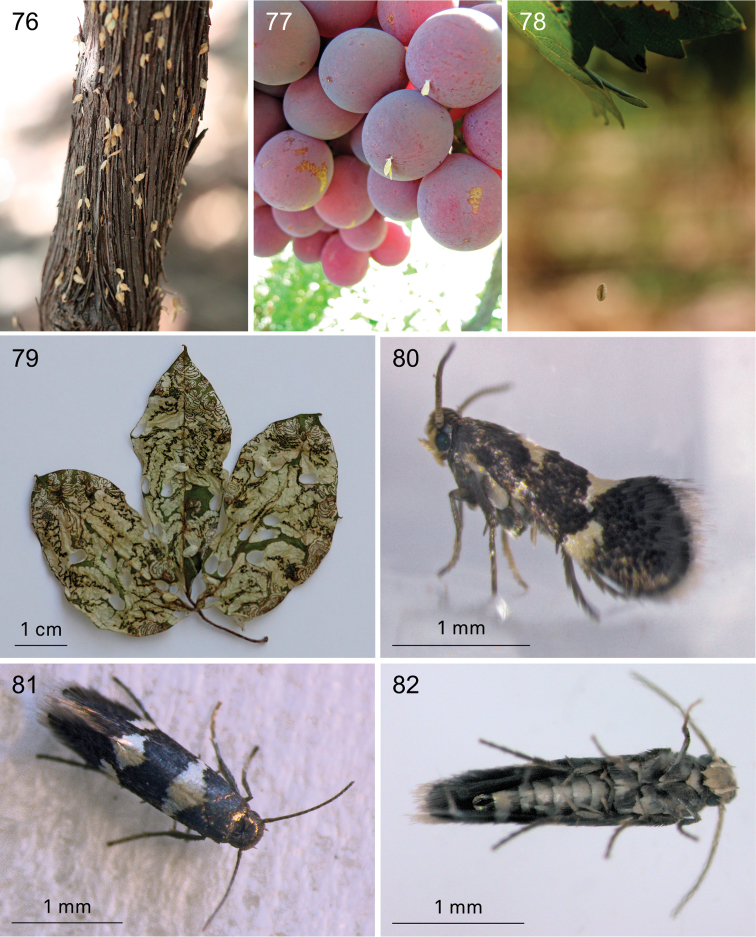
*Holocacista capensis*, life history. **76** Trunk of *Vitis* with many cocoons with exuviae, De Anker, Paarl, 14 February 2013 **77** grapes with fresh cocoons attached, Paarl, 25 January 2013 **78** larva in cocoon, going down on silken thread, Paarl, 16 January 2013 **79** Leafmines in *Rhoicissus digitata*, reared in laboratory from adults that originated on *Vitis* from Wellington, 2014 **80–82** Live adult males, reared from *Vitis vinifera*, from Paarl **80** EvN2013004, 28 January **81** Windmeul, reared in Leiden, 23 February 2012 **82** EvN2013005, 4 February.

**Figures 83–88.**
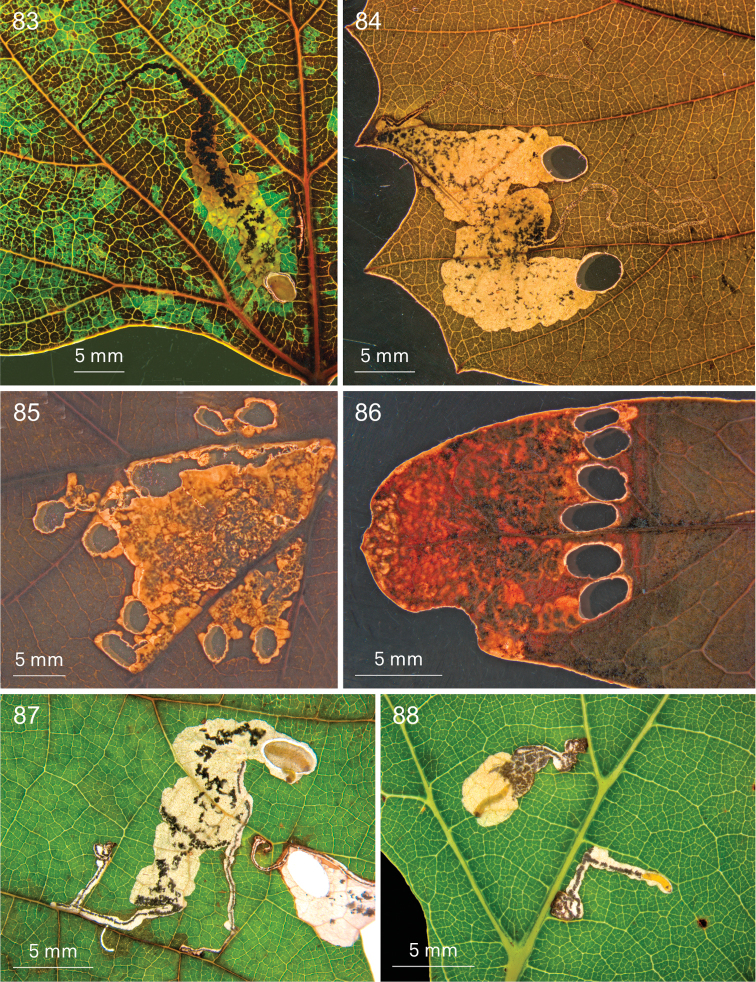
*Holocacista* species, leafmines on Vitaceae. **83** *Holocacista* sp. *Rhoicissus_tomentosa*, fresh mine, larva cutting out shield, Swellendam, EvN2014901, larva RMNH.INS.30313 **84** *Holocacista* sp. *Rhoicissus_tridentata*, dried mines, Vari 1225 **85** *Holocacista salutans* on *Rhoicissus tomentosa*, dried mines, Vari 2788 **86** *Holocacista salutans* on *Rhoicissus digitata*, dried mines, Vari 3342 **87, 88** *Holocacista rivillei*, fresh mines with larvae on *Vitis vinifera*, in 87 two larvae next to each other, one in cocoon, EvN2013904.

**Figures 89–93.**
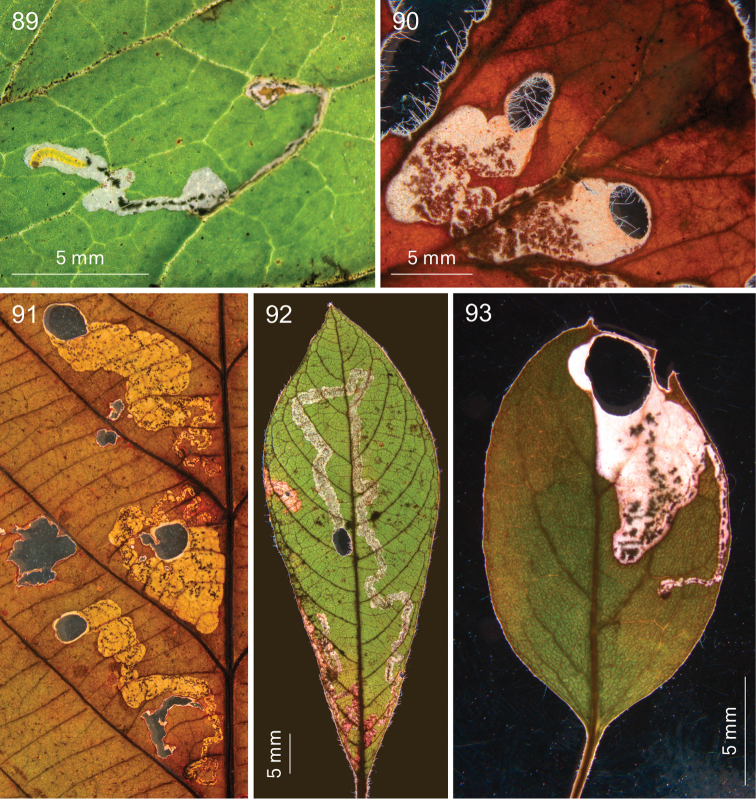
*Holocacista* species, leafmines on various plants **89** *Holocacista varii*, fresh mine with larva on *Pelargonium*, EvN2013033 **90** *Holocacista varii*, dried mine on *Pelargonium cucullatum*, EvN2013021 **91** *Holocacista* sp. *Leea_*Borneo, dried mines on *Leea indica*, EvN2005252 **92** *Holocacista* sp. *Lasianthus_*Borneo, dried mine on *Lasianthus* sp., EvN2005255 **93** *Holocacista* sp. *Paederia_*Taiwan, dried mine on *Paederia*, EvN2012314.

**Figures 94–100.**
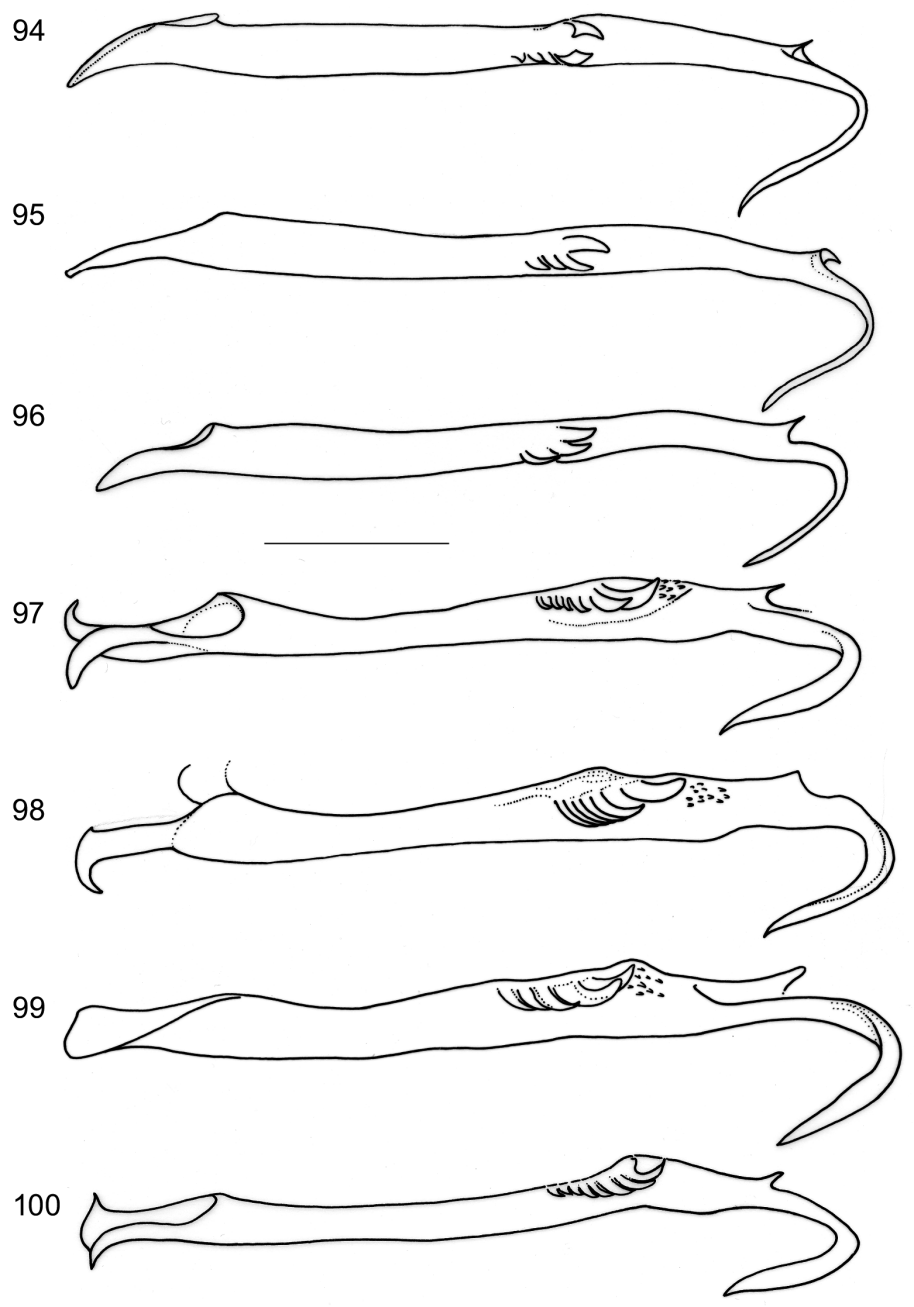
*Holocacista* species, male phallus in lateral view, scale 100 μm. **94–96** *Holocacista capensis*, Genitalia slides resp. EvN4264, EvN4446 [mirrored], EvN4381 **97–100** *Holocacista salutans*, Genitalia slides resp. TM4023 [type locality], EvN4383, EvN4384. All on same scale.

**Figures 101–106.**
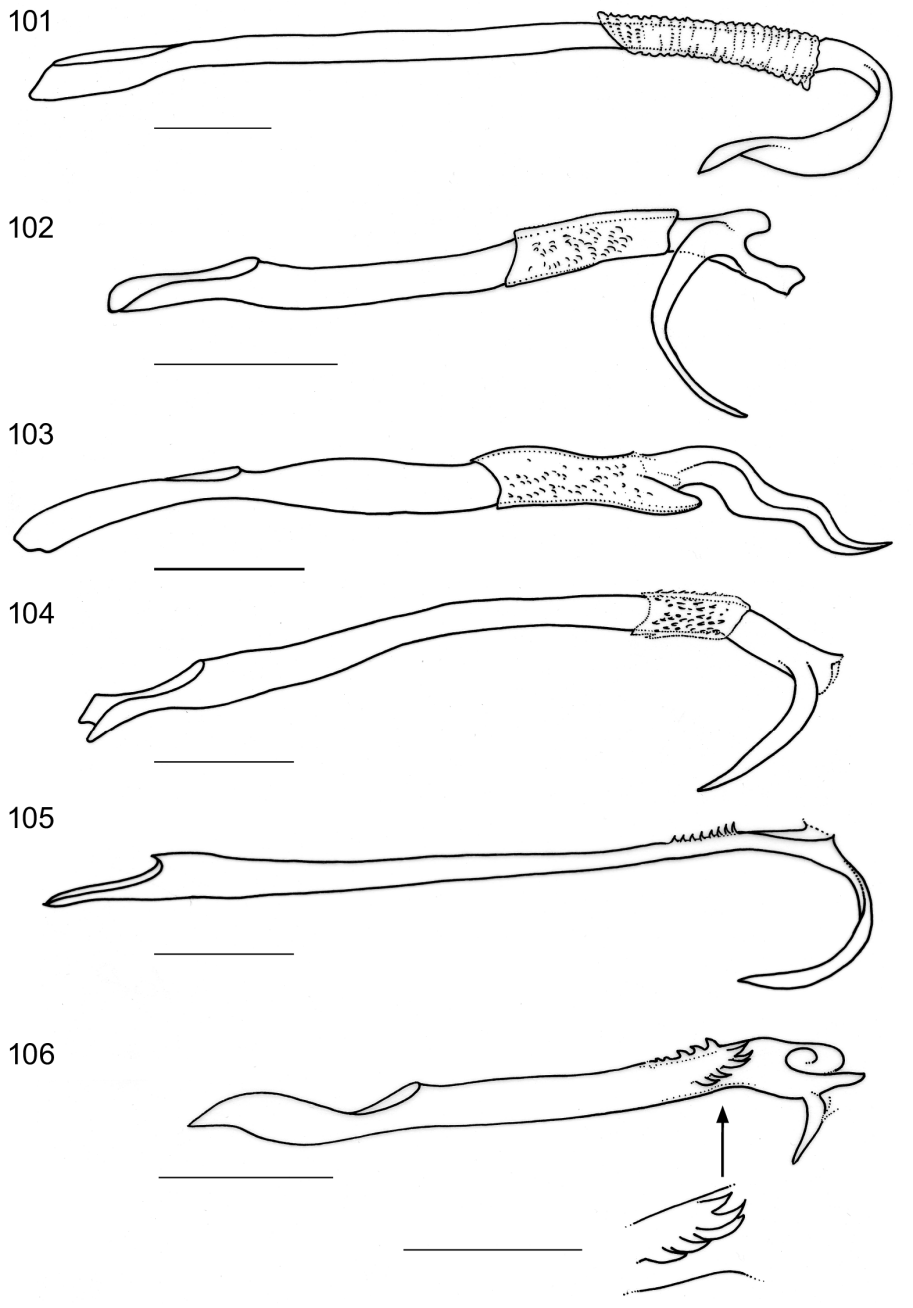
*Holocacista* species, male phallus in lateral view, scales 100 μm. **101** *Holocacista* sp. *Rhoicissus_tridentata*, Genitalia slide EvN4380 [mirrored] **102** *Holocacista* sp. *Rhoicissus_*PundaMilia, Genitalia slide EvN4382 **103** *Holocacista* sp. *Cissus_integrifolia*, Genitalia slide EvN4387 **104** *Holocacista varii*, Genitalia slide EvN4623 **105** *Holocacista rivillei*, Genitalia slide EvN4443 [mirrored] **106** *Holocacista* sp. *Dyerophytum_*UAE, Genitalia slide EvN4628. 104 and 105 on the same scale.

**Figures 107–114.**
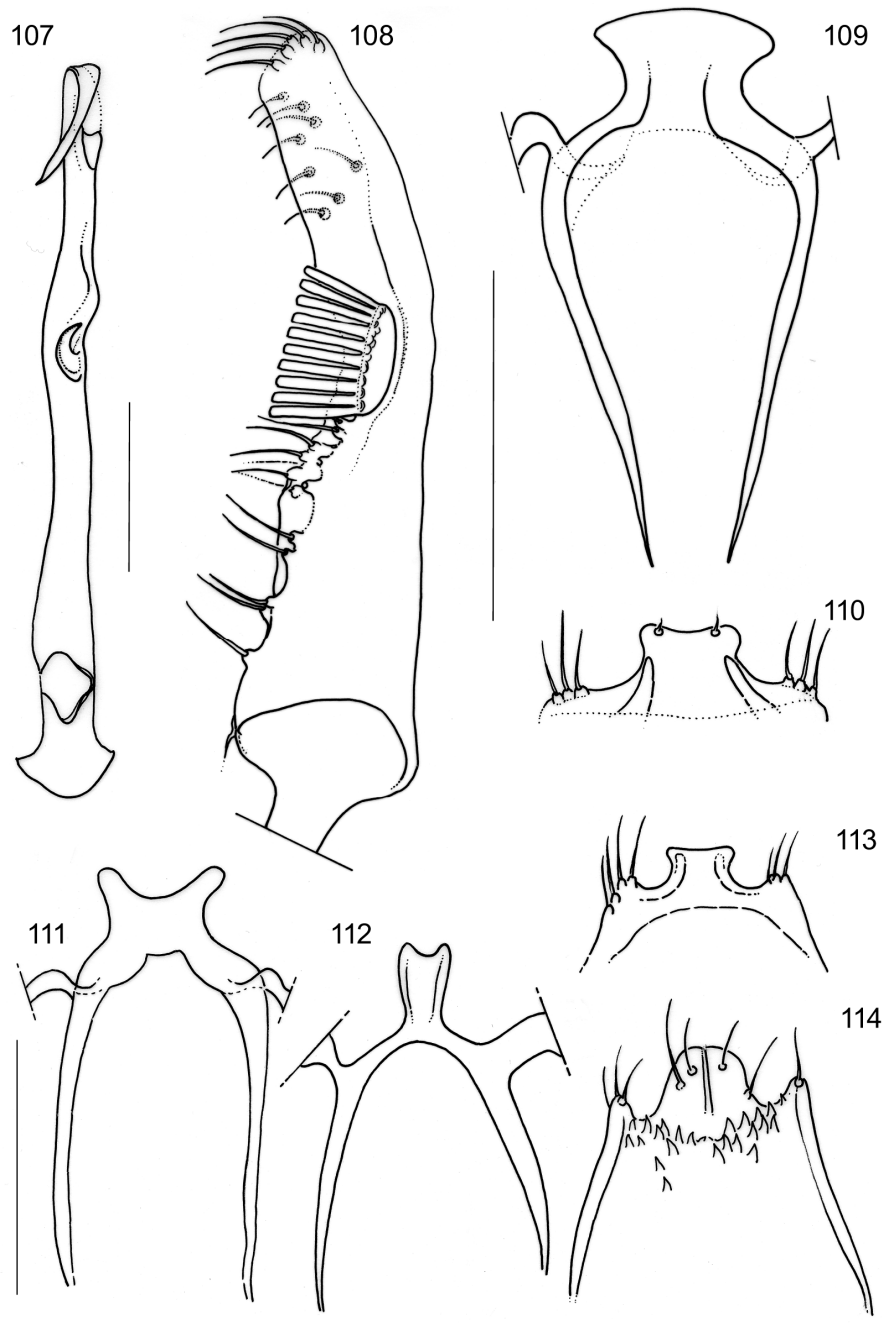
*Holocacista* species, male genitalia, details, scales 100 μm. **107–110** *Holocacista capensis*, Holotype, Genitalia slide EvN4622 **111, 113** *Holocacista salutans*, Genitalia slide EvN4383 **112, 114** *Holocacista varii* Genitalia slide EvN4623 108–110, same scale; 111–114 same scale.

**Figures 115–116.**
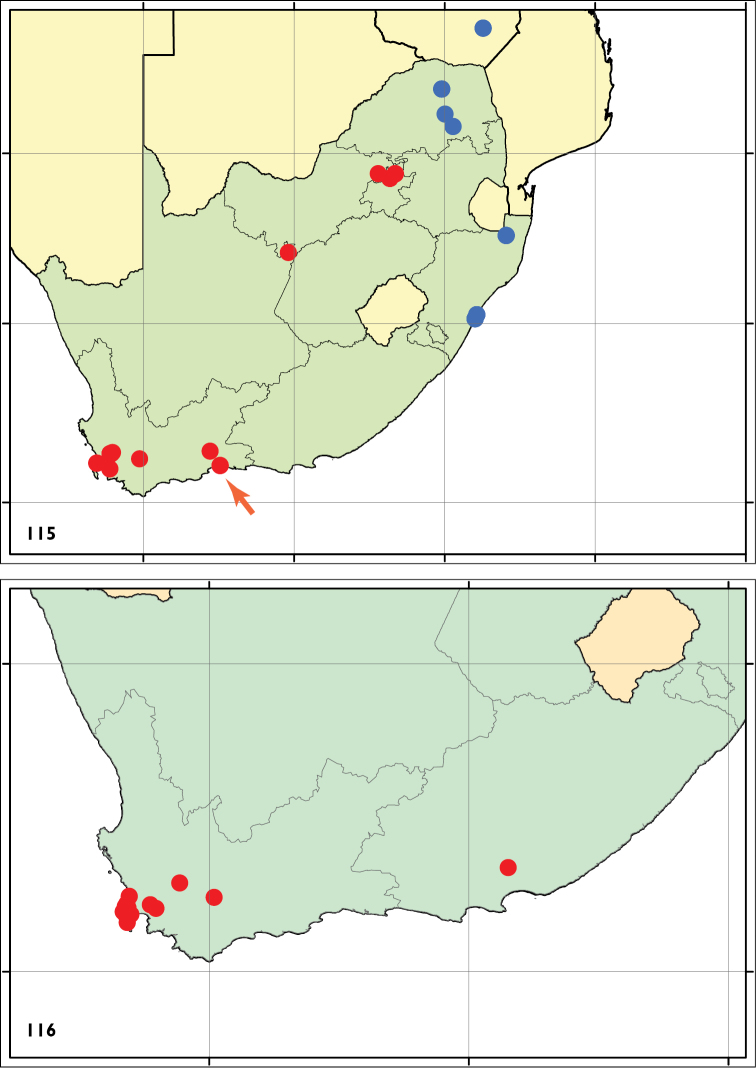
Distribution records of southern African *Holocacista* species. **115** *Holocacista capensis* (red dots) and *Holocacista salutans* (blue dots); arrow points at Wilderness, only locality where *Holocacista capensis* was found on native *Rhoicissus digitata* **116** *Holocacista varii*.

### DNA barcoding and species relationships of *Holocacista capensis* Barcode analysis

**Figure 117.**
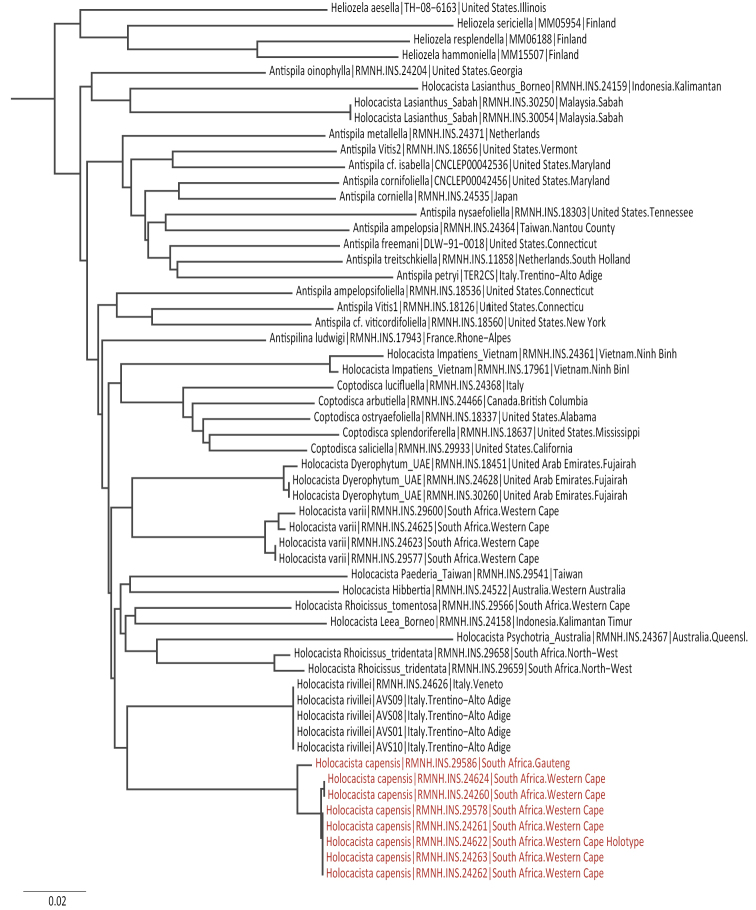
Neighbor Joining Tree, based on DNA barcodes of *Holocacista* species and other Heliozelidae.

We sequenced eight specimens of *Holocacista capensis* and compared those with 24 sequences of other *Holocacista* species and several other heliozelid barcodes, sequenced for previous studies (van Nieukerken et al. 2012b; Bernardo et al. 2015). The NJ tree is presented in Fig. 117. The barcodes of *Holocacista capensis* group clearly together and can only be divided in three haplotypes, two from the Paarl region, which just differ in a single substitution on position 59 (C or T), whereas the single sequenced specimen from Gauteng has a difference of 1.4% (9 nucleotides). The nearest neighbours are a specimen of *Holocacista varii* and a specimen of *Holocacista rivillei*, each with a distance of 11.2%. The three sequenced larvae from *Rhoicissus* represent two barcode clusters: respectively the two larvae from *Rhoicissus tridentata* in North West province (12.0–12.2% distance from *Holocacista capensis*), and the single sequence of a larva from *Rhoicissus tomentosa* in Western Cape (11.9% distance). We failed in amplifying DNA from collection specimens of South African Heliozelidae stored in TMSA, also when using primers for smaller parts of the CO1 gene.

Most *Holocacista* barcodes group together in the NJ tree, but a few Asian ones cluster at different parts of the tree.

### Infestation of Grapevine in South Africa by *Holocacista capensis*

Since its initial discovery in 2011 on table grape vines in Paarl, the moth is now reported from Cape Town, Somerset-West, Stellenbosch and Wellington; in all cases reports were in conjunction with damage reported on the foliage of table grape or wine grapevines. Various cultivars have been affected, but no single preference for a particular cultivar was obvious. Infestations, as indicated by the number of mines per leaf or descending larvae from the canopy, range from small to extensive. Thus far, no reports have been received of its presence in the Hex River Valley, the major table grape producing region of South Africa, although a report has been received from Robertson, close to that region.

The very first record of its presence on vines now appears to be that of Dr Lajos Vári, breeding the moth from an urban grape vine in his own garden in Pretoria. In 1980, moths were bred in Northern Cape, Vaalhartz Research Station and later, infested vine leaves from Roodeplaat yielded moths in 1990. The first records from the Western Cape were moths, bred from infested urban vine foliage collected in March 1998 from Oudtshoorn. Although it was first noticed in commercial table grape vines in 2011 in the Western Cape, field observations indicate that it must have occurred much earlier in the region on grape vines. All collections made of leafmines in 2011 to date, have yielded larval or pupal parasites, an indication of a longer presence of the moth. Reports from field workers indicated that they had noticed the scale-like larval or cocoon shields earlier on grape vines, but were then of the opinion that these were either discarded scale insect exuviae or plant debris.

At present, the moth is widespread in the grape vine regions of the Western Cape, but mainly restricted to that region south of the Cape Fold Mountain range, although one record is known from Robertson, north of that range. In the northern part of South Africa, it occurs in high population numbers in the table grape region at Brits, east of Pretoria. Records also exist of its occurrence in the Vaalhartz region (Northern Cape) and in Oudtshoorn (Western Cape).

The effect of the leafmines on the grape vine itself appears to be limited. At the height of the moth season, most table grape vines are under irrigation and lost leaf growth is quickly replaced with new foliage throughout the growing season. Collateral damage by the larvae, when at high population numbers, may be more serious, especially when larvae descend from the vine canopy to form a dense curtain of suspended larvae. This not only harasses the harvesters themselves and contaminates the grape bunches, but in some cases, the radiators of tractors collecting the grape bunches became completely blocked by this curtain (in one case, this occurred in a distance of less than 200 m), requiring constant removal of the larvae.

## Discussion

### Taxonomy and identification

As in previous studies on unexpected infestations of grapevine and walnuts by Heliozelidae (van Nieukerken et al. 2012b; Bernardo et al. 2015), the lack of a taxonomic framework of this family made the identification difficult, and required a detailed taxonomic study. For Africa, the situation was in fact worse than in the cited examples from North-America, where at least an extensive literature on leafminers exists. Here only a single species had been described in recent years according to modern taxonomic methodology, with details on genitalia (Mey 2011); in addition to two very short – now useless – descriptions nearly a century old. Only by detailed study of material borrowed from the collections in Pretoria could we reach the conclusion that our species was unnamed and belongs to the genus *Holocacista*, which was hitherto only known from a single Mediterranean species. Thanks to the long lasting efforts of the former curator of the Transvaal Museum, Lajos Vári, who reared and collected leafminers from many different species throughout southern Africa between 1950 and 1990, there was a firm basis available for this study in the collections in Pretoria, even though nothing on this particular group had been published previously. We cannot stress enough the importance of maintaining and enlarging such collections for studies such as the present one.

While redescribing the genus *Holocacista*, we could also assign various other South African, Asian and Australian species to the genus. This further showed that also in those areas the family Heliozelidae has been poorly studied.

Unfortunately, the failure of amplifying DNA from the South African collection specimens has prevented a comparative usage of DNA barcodes. Our research has also shown that still many unnamed species exist, and additional revisionary taxonomic work on this fauna and on other leafminers, including fieldwork, is highly important, where the possibility exists that this and other economic important plants could become infested.

### Origin and host shift of the grapevine miner

In our study we were only able to associate the new species with a single population feeding on native *Rhoicissus digitata*. Up to now we do not have any match yet of DNA barcodes of native *Rhoicissus* feeders, of which we sequenced two possible (new) species. Interestingly, the only *Rhoicissus*-feeding population of *Holocacista capensis* has been found in the Western Cape, from where also the majority of the grapevine miner records originate. This could be an indication that the host shift from *Rhoicissus* to *Vitis* may have taken place in the Western Cape. We assume that in the past the natural vegetation with *Rhoicissus* was much more widespread and more often close to vineyards, making such a shift far easier. However, this would mean that the shift may have taken place more than a century ago or even longer. Grapevine has been grown in the Western Cape since the Dutch settled at the Cape in the 17^th^ century (http://en.wikipedia.org/wiki/History_of_South_African_wine). If that is the case, the miner has apparently been able to occur in low densities without being spotted before Lajos Vári recorded it first from his own garden in Pretoria in 1950 (this study). The occurrence in Gauteng and the Northern Cape could in that scenario be explained by a later infestation from the Western Cape. The Paarl-Wellington region is the major cultivation area of vine nursery stock in South Africa. Major cultivars are propagated and dispatched in large quantities to commercial farms and nurseries throughout the country. Rootstock in the form of hardwood cuttings, harvested during late autumn and winter, is obtained from a variety of sources before being grafted with the desired cultivar. This may explain the infestation of other areas with the moth. Visits to home garden nurseries in the Western Cape detected many grape vines being infested with the leafminer.

The 1.4% barcode difference between the single sequenced Gauteng specimen and the Paarl specimens could be an indication for a genetic variation between the Gauteng and Western Cape populations, although the genetic variation in a single population of *Holocacista varii* that we observed is in fact larger. Still, genetic variation could have been caused by repeated host shifts from *Rhoicissus* to *Vitis* from genetically different populations. The fact that we easily could rear offspring of *Vitis* reared adults onto *Rhoicissus digitata* also supports this hypothesis. This would mean that the barcode variation represents the original variation of this species on its native hosts. This is not so unlikely, since several of the Vitaceae-feeding Heliozelidae appear to have wider host ranges than a single genus, and may occur on e.g., both *Vitis* and *Parthenocissus*, such as *Antispila oinophylla* (van Nieukerken et al. 2012b); whereas in that paper we reported this double host association only from Italy, it has since also been observed in North America (J.F. Landry, personal communication), or sharing *Vitis* and *Ampelopsis* in *Antispila ampelopsia* Kuroko (Kuroko 1961). In Japan a comparative shift from native to cultivated hosts also occurred, but then within the genus *Vitis*: *Antispila uenoi* Kuroko shifted from native *Vitis coignetiae* Pulliat ex Planch. to the cultivated North American *Vitis labrusca* L. and became a pest (Kuroko 1987; Ueno et al. 1987). However, within a rather uniform genus such as *Vitis*, such a shift is hardly surprising, since most species share leaf structure and chemistry, and usually also share the same herbivores. It seems that a shift from *Rhoicissus* to *Vitis* would require more adaptation; whereas most *Rhoicissus* are evergreen, *Vitis* is deciduous, and in South Africa its leaves fall in May. Moths that emerge late will not be able to find an oviposition place and will perish. Our observations during the winter seasons of 2012–2013 show that moths only start emerging from early September onwards in synchrony with the appearance of grapevine foliage. It is possible that the life history of the native moths feeding on *Rhoicissus* in the Western Cape was already synchronised with the cooler winter climate, and therefore emergence of moths was rare in the winter. Another indication for this could be that the closely related *Holocacista* species in the Highveld of Gauteng also hibernate in their cocoons during the cold winter months. However, differences in leaf texture between *Rhoicissus* and *Vitis* may require further adaptation, although it is possibly easier for a species that feeds on tougher leaves to adapt to softer leaves than vice versa.

Another case of host shift by a heliozelid is the recent infestation of walnut in Italian orchards by the North American *Coptodisca lucifluella* (Bernardo et al. 2015). It seems that this species shifted host from its original American host, the genus *Carya*, to *Juglans*, possibly after its introduction.

Where the origin of the grapes planted in South Africa is almost universally European (http://en.wikipedia.org/wiki/History_of_South_African_wine), it is not very likely that *Holocacista capensis* originated somewhere other than in South Africa. Its close relationship to several other South African Vitaceae miners makes it very plausible that *Holocacista capensis* indeed is a native South African insect. For tracing the origin of the host shift, a larger scale inventory of South African Vitaceae miners with detailed DNA analysis is required.

There are only few other insects known that feed both on *Rhoicissus* and *Vitis*, but at least two hawk moths (Sphingidae) are reported from both genera: *Hippotion celerio* (Linnaeus, 1758), that is more polyphagous (in the Western Cape during the winter months its larvae feed on *Zantedeschia aethiopica* (L.) Spreng.), and *Theretra capensis* (Linnaeus, 1764), that seems to be specialised on Vitaceae (Kroon 1999). However, external-feeding large caterpillars, such as these hawk moths, require different adaptations compared to leafminers. On *Rhoicissus* we also noted leafmines belonging to the genus *Phyllocnistis* Zeller, 1848 (Gracillariidae) that may belong to an undescribed species.

### Infestation of vineyards and table grape plantations

It is apparent that *Holocacista capensis*, as shown by the presence of larval and pupal parasites, must have been present for some time in the vineyards in this region. Only when moth densities reached alarmingly high numbers, was identification called for. Although damage by the larvae to the grapevine foliage itself is limited, the reported collateral damage by the larvae can be serious. Also, contamination of grapes with cocoons is cumbersome, since they need to be removed manually before the grapes can be sold or exported.

A first step needed to control the insects is assessing its density, by measuring the number of mines per leaf, infested leaves per vine, and particularly by setting out pheromone traps (Wang et al. 2015). This should be organised in selected vineyards in the vine growing regions of the greater Western Cape. Currently, already some of this research is planned.

Control of the moth itself appears to be difficult. It appears that all cultivars of table grapes, especially those grown under a dense canopy cover, are attacked; wine grape cultivars are not that seriously affected and the mines are of little concern to the wine producer, but the moth can maintain populations in the vineyards that could infest table grape plantations. Moth densities are highest at the time of table grape harvest, with the result that no chemical control is feasible. Larvae are well protected within the leaf itself, ruling out the use of a systemic insecticide at the time when grape berries are developing and ripening. Although parasitoids are present, their numbers are low and only reach higher numbers near the end of the grape season. Ants could be efficient predators, but their numbers are controlled by the vine farmers. In two cases, numbers of moths were drastically reduced when insecticides were applied for the control of some other pests during October-November, the start of the vine growing season; investigations on this aspect are at present being carried out. Another measure that resulted in much lower densities was manual removal of cocoons from trellises and trunks during winter, but this is a time consuming method. Further research is also needed to see if mating disruption with pheromones is a serious possibility.

## Supplementary Material

XML Treatment for Holocacista

XML Treatment for Holocacista rivillei

XML Treatment for Holocacista capensis

XML Treatment for Holocacista salutans

XML Treatment for Holocacista varii

## Appendix A

**Southern African Heliozelidae**

The following tentative checklist and short diagnoses are based on the collections in the Ditsong Museum in Pretoria, mostly gathered during extensive rearing of leafmines by Lajos Vári and a few other records, including our own. For most Vitaceae feeders some genitalia have been dissected, and for all taxa adults and leafmines have been examined. In all, 12 species are recognized, but differences in mines and variation in genitalia suggest there are more. Additionally, even more may be expected in parts of southern Africa where Vári rarely collected. A revision of this group should preferably include collecting of new material and molecular analyses.

**Genus *Heliozela* Herrich-Schäffer, 1853**

***Heliozela argyrozona* (Meyrick, 1918), comb. n.**

*Antispila argyrozona* Meyrick, 1918: 35. Holotype ♂: South Africa, [KwaZulu Natal], Eshowe, 4.i.[19]16, A.J.T. Janse; “29 51”; Type No. 108 (TMSA) [examined].

Wingspan ca. 4.0–4.5 mm. Uniformly dark bronze, male with a white medial costal spot, female with a medial fascia and usually a small costal spot at 1/3. A typical *Heliozela*, with distinct epiphysis and complex venation. Assignment of the reared series to *Heliozela argyrozona* is tentative and based on external resemblance of the females with the holotype; no genitalia have been examined.

**Hostplant.** *Syzygium cordatum* Hochst. ex Krauss (Myrtaceae). Mine a sinuous gallery on leaf upperside, gradually becoming wider.

**Distribution.** Kwazulu-Natal, Limpopo.

**Material examined. South Africa**: 34 adults, mines on 10 leaves, Limpopo, Louis Trichardt, 6–8.v.1953, Ac. no. 740, 743, leafmines on *Syzygium cordatum*, emerged 2.vi.–4.ix.1953, L. Vári, Genitalia slide TM3825; 1 adult, KwaZulu-Natal, Umdoni park, 15.v.1974, Ac. no. 3286, leafmines on *Syzygium*, emerged 17.vii.1974, L. Vári.

***Heliozela* sp. 2**

Resembles *Heliozela argyrozona*, but has two fasciae.

**Hostplant.** unknown.

**Distribution.** Limpopo.

**Material examined. South Africa**: 1 ♀, Limpopo, Woodbush, 14–16.ix.1960, van Son & Vári.

**Genus *Antispila* Hübner, 1825**

***Antispila* sp.**

This is the largest South African heliozelid with a wingspan of 7–7.5 mm and large yellowish fascia and spots (Fig. 17). The antennae are distinctly ringed and have 26 segments. The male has a conspicuous large patch of yellow and black androconial scales on the forewing and hindwing underside.

**Hostplant.** *Rhoicissus rhomboidea* (Vitaceae). Mines are conspicuous large blotches and have a large cut out of about 6 mm long, which separates them easily from the *Holocacista* mines.

**Distribution.** Eastern Cape, Limpopo. Only three specimens and three herbarium sheets in TMSA.

**Material examined. South Africa**: 1 moth, 1 sheet with mines, Eastern Cape, Grahamstown, 2.xii.1954, Ac. no. 1471, leafmines on *Cissus rhomboideus*, emerged 30.xii.1954, L. Vári; 1♂, 1♀, 2 sheets with mines, Limpopo, Louis Trichardt, 2.v.1953, Ac. no. 674, leafmines on *Cissus rhomboideus*, emerged 14.vii–30.viii.1953, L. Vári, Genitalia slide ♂ EvN4379.

**Genus *Holocacista* Walsingham & Durrant, 1909**

***Holocacista capensis* van Nieukerken & Geertsema, sp. n.**

See above.

***Holocacista salutans* (Meyrick, 1921), comb. n.**

See above.

***Holocacista varii* (Mey, 2011), comb. n.**

See above.

***Holocacista* sp. *Rhoicissus_tridentata***

Wingspan ca. 4.0–4.8 mm. Differs from *Holocacista capensis* and *Holocacista salutans* by smoother bronze scaling, costal and tornal spots about same size, antennae hardly ringed, fringe line not pronounced. In male genitalia very long phallus, without spines, but a folded sleeve (Fig. 101).

**Hostplant.** Rhoicissus tridentata subsp. cuneifolia (Vitaceae). Possibly also on *Rhoicissus rhomboidea*, and *Rhoicissus tomentosa*, but this needs to be verified. Mines characterised by long initial linear gallery filled with frass (Fig. 84). Larvae from October to June, those from May and June hibernate and moths emerge in September and October.

**Distribution.** Gauteng, KwaZulu-Natal, North West, Zimbabwe: Harare.

**Remarks.** The majority of specimens reared from *Rhoicissus tridentata* leafmines, as listed below are probably this species as characterised by the described externals, phallus and leafmine, but at least *Holocacista salutans*, or a closely related species and *H. Rhoicissus_PundaMilia*, also occur on this host and there may be more hidden in this material. Vári used at least three manuscript names in his notebooks for specimens from this host, indicating he also considered not all to belong to one species: “*Antispila denticola*,” “*Antispila cuneifoliella*” and “*Antispila hartwigi*”. We associate tentatively the two DNA barcodes with this species, but this requires confirmation.

**Material examined. South Africa, Gauteng**: 7 adults, 2 sheets with mines, Pretoria, Wonderboom Zuid, 1.iv.1950, Ac. no. 298, leafmines on *Rhoicissus tridentata*, emerged 12.x–30.xi.1950, L. Vári, Genitalia slides ♂ TM6767, ♀ TM6769; 12 adults, 1 sheet with mines, ibidem, 16.xii.1950, Ac. no. 298, emerged 29.xii.1950–10.i.1951; 26 adults, 2 sheets with mines [mixed with mines of Holocacista cf. salutans, Pretoria, Meintjeskop, 7.vi.1951, Ac. no. 339, 340, 341, leafmines on *Rhoicissus tridentata*, emerged 19.ix–9.x.1951, L. Vári, Genitalia slides TM6768, TM6770, ♀ TM6829, ♂ TM10347; 17 adults, ibidem, 27.v.1953, Ac. no. 833, emerged 27.ix–6.xi.1953; 1♂, 1♀, Suikerbosrand, 21–24.x.1975, Ac. no 3472, leafmines on *Rhoicissus* sp., emerged 24.x–10.xi.1975, M.J. Scoble. **KwaZulu-Natal**: 4 adults, 1 envelope with leafmines, Mt. Edgecombe, 31.iii.1954, Ac. no. 1225, leafmines on *Rhoicissus tridentata*, emerged 21–26.iv.1954, L. Vári, Genitalia slide ♂ EvN4380. **North-West**: 2 larvae (RMNH.INS.29658, 29659), Kgaswane Nature Reserve, Rustenburg, 27.i.2013, EvN2013901, leafmines on *Rhoicissus tridentata*, M. Stiller. **Zimbabwe**, **Harare**: 1♂, Mt. Selinda, 8.iv.1956, Ac. no. 1784, leafmines on *Rhoicissus tridentata*, emerged 29.iv.1956, L. Vári, Genitalia slide EvN4385.

***Holocacista* sp. *Rhoicissus_tomentosa***

We have no adults that we can associate with certainty with the distinctive mine of this species. We barcoded two larvae (RMNH.INS.29566, 30313). The samples with leafmines from the Vári collection often show several mine types, apart from this species also including *Holocacista salutans* and possibly the previous species or others. Further work is needed to differentiate these taxa.

**Hostplant.** *Rhoicissus tomentosa* (Vitaceae). Mine a gallery, first filled with frass, later an elongate gallery-like blotch with central clumped frass (Fig. 83).

**Distribution.** Limpopo, Western Cape, Zimbabwe: Harare.

**Material examined. South Africa, Limpopo**: ??? adults, 2 sheets with mines (mixed), Louis Trichardt, 17.iii.1964, Ac. no. 2694, leafmines on *Rhoicissus tomentosa*, adults emerged 7–10.iv.1964, L. Vári. **Western Cape**: 1 larva, RMNH.INS.29566, leafmines, Swellendam E, Zuurbraak, 17.i.2013, EvN2013007, leafmines on *Rhoicissus tomentosa*, E.J. van Nieukerken & H. Geertsema; 1 larva, RMNH.INS.30313, leafmines, ibidem, 16.vi.2014, EvN2014009, H. Geertsema. **Zimbabwe, Harare**: 1 adult?, 1 sheet with mines, Mt. Selinda, 8.iv.1956, Ac. no. 1783, leafmines on *Rhoicissus tomentosa*, emerged 4.v.1956, L. Vári. **Zimbabwe, Masvingo**: 1 sheet with mines, Vumba, Mareh, 7.iii.1964, Ac. no. 2649, L. Vári.

***Holocacista* sp. *Rhoicissus_*PundaMilia**

Wingspan 3.0–4.0 mm, very small species. Externally inseparable from *Holocacista salutans*. Male genitalia characterised by very different, recurved phallus appendage (Fig. 102).

**Hostplant.** *Rhoicissus digitata* and Rhoicissus tridentata subsp. cuneifolia (Vitaceae). Mines a narrow gallery with medial linear frass, gradually enlarging into a wide gallery, rather different from those of *Holocacista capensis* and *salutans* on *Rhoicissus digitata*.

**Distribution.** Gauteng, Limpopo.

**Material examined. South Africa, Gauteng**: 1♂, Pretoria, Meintjeskop, 7.vi.1951, Ac. no. 339, leafmines on *Rhoicissus tridentata*, emerged 8.x.1951, L. Vári, Genitalia slide TM6828. **Limpopo**: 2 adults, Punda Milia, K.N.P. Survey [Kruger National Park Survey], 23.xi.1961, Ac. no. 2362, leafmines on *Rhoicissus digitata*, emerged 11.xii.1961, L. Vári; 5 adults, 1 sheet with 10 mines, ibidem, 4.xii.1964, Ac. no. 2712, leafmines on *Rhoicissus digitata*, emerged 21.xii.1964, Genitalia slide EvN4382.

***Holocacista* sp. *Cissus_integrifolia***

Wingspan 3.5–4.0 mm, small species. Externally similar to *Holocacista* sp. *Rhoicissus_tridentata*, with bronze wings, and antennae not ringed. Male genitalia characterised by the phallus appendage that is not curved backwards, but sinuous (Fig. 103).

**Hostplant.** *Cissus integrifolia* (Vitaceae). Mines very compact, start with spiral gallery, later becoming an elongate blotch with frass in zigzag pattern.

**Distribution.** Zimbabwe: Masvingo.

**Material examined. Zimbabwe, Masvingo**: 11 adults, 1 sheet with 15 mines, Lundi, Lundi river, 4.iii.1964, Ac. no. 2646, leafmines on *Cissus integrifolia*, emerged 21–25.iii.1964, L. Vári, Genitalia slide ♂ EvN4387.

***Holocacista* sp. *Lannea_*SA**

Wingspan 4–5 mm. Wings bronze fuscous with shining fascia and costal and tornal spots, rather similar to *Holocacista* sp. *Rhoicissus_tridentata*. Antennae not ringed. Genitalia not examined.

**Hostplant.** *Lannea discolor*, *Lannea* sp. (Anacardiaceae). Mine very compact, somewhat resembling those of *Holocacista capensis*. Initial gallery with zigzag turns, closely to each other, later completely enclosed in blotch.

**Distribution.** Gauteng.

**Remarks.** Vári gave the manuscript name “*Antispila lanneivora*”.

**Material examined. South Africa, Gauteng**: 1♂, Pretoria, behind garden Prof. J[anse], 16.i.1951, Ac. no. 317, leafmines on *Lannea discolor*, emerged 2.ii.1951, L. Vári; 4 adults, 1 sheet with 12 mines, Pretoria, above Pierneef St., 16.i.1951, Ac. no. 618, leafmines on *Lannea discolor*, emerged 9–12.ii.1951, L. Vári; 5 adults, Pretoria, Magalies Mountain, 26.xii.1953, Ac. no. 1016, leafmines on *Lannea* sp., emerged 8–11.i.1951, L. Vári.

***Holocacista* sp. *Terminalia_*SA**

Wingspan 3.0–3.7 mm, very small moths. Wings greyish or brown with narrow medial fascia. Venation extremely reduced, forewing with 4 veins only, Rs, R+M, CuA and A.

**Hostplant.** *Terminalia prunioides* (Combretaceae). Mine a narrow gallery, suddenly enlarging into a roundish blotch.

**Distribution.** Namibia: Erongo

**Remarks.** Placement in *Holocacista* is tentative.

**Material examined. Namibia, Erongo**: 2 adults, 2 leaves with 2 mines, Karabib, 20.v.1959, Ac. no. 2108, leafmines on *Terminalia prunioides*, emerged 15.vi.1959, L. Vári, Genitalia slide ♂ EvN4387.

## Supplementary materials

Supplementary material 1 All material examined and used for DNA barcoding Data type: Excel spreadsheet (xlsx). Explanation note: The dataset comprises all material examined and identified to a certain extent for this study. All specimens used for the descriptions of genus and listed species are included, as are all DNA barcoded specimens, including those that were published earlier. This dataset is made available under the Open Database License (http://opendatacommons.org/licenses/odbl/1.0/). The Open Database License (ODbL) is a license agreement intended to allow users to freely share, modify, and use this Dataset while maintaining this same freedom for others, provided that the original source and author(s) are credited.Erik J. van Nieukerken, Henk Geertsema
